# Resistance of HNSCC cell models to pan-FGFR inhibition depends on the EMT phenotype associating with clinical outcome

**DOI:** 10.1186/s12943-024-01954-8

**Published:** 2024-02-21

**Authors:** Felix Broghammer, Irina Korovina, Mahesh Gouda, Martina Celotti, Johan van Es, Inga Lange, Cornelia Brunner, Jovan Mircetic, Robert P. Coppes, Olivier Gires, Andreas Dahl, Michael Seifert, Nils Cordes

**Affiliations:** 1 OncoRay - National Center for Radiation Research in Oncology, Faculty of Medicine Carl Gustav Carus, Technische Universität Dresden, 01307 Dresden, Germany; 2 Institute of Radiooncology - OncoRay, Helmholtz-Zentrum Dresden-Rossendorf (HZDR), 01328 Dresden, Germany; 3 Department of Otorhinolaryngology, Head and Neck Surgery, Ludwigs-Maximilians-University University Hospital, 81377 Munich, Germany; 4 Hubrecht Institute, Royal Netherlands Academy of Arts and Sciences (KNAW) and University Medical Center Utrecht, 3584 CT Utrecht, the Netherlands; 5 Department of Otorhinolaryngology, Ulm University Medical Center, 89075 Ulm, Germany; 6 German Cancer Consortium, Partner Site Dresden: German Cancer Research Center (DKFZ), 69120 Heidelberg, Germany; 7 Mildred Scheel Early Career Center (MSNZ) P2, Medical Faculty and University Hospital Carl Gustav Carus, Technische Universität Dresden, 01307 Dresden, Germany; 8 Department of Biomedical Sciences of Cells and Systems, Section of Molecular Cell Biology, University Medical Center Groningen, University of Groningen, 9713 Groningen, The Netherlands; 9 Department of Radiation Oncology, University Medical Center Groningen, University of Groningen, 9713 Groningen, The Netherlands; 10 DRESDEN-Concept Genome Center, Center for Molecular and Cellular Bioengineering, Technische Universität Dresden, 01307 Dresden, Germany; 11 Institute for Medical Informatics and Biometry (IMB), Faculty of Medicine Carl Gustav Carus, Technische Universität Dresden, 01307 Dresden, Germany; 12 National Center for Tumor Diseases (NCT), Partner Site Dresden, German Cancer Research Center (DKFZ), 69192 Heidelberg, Germany; 13 Department of Radiotherapy and Radiation Oncology, University Hospital Carl Gustav Carus, 01307 Dresden, Germany

**Keywords:** HNSCC, Fibroblast growth factor receptor, Epithelial-to-mesenchymal transition, Epidermal growth factor receptor, Radiosensitization, Radioprotection, Adaptive resistance, β1 integrin

## Abstract

**Background:**

Focal adhesion signaling involving receptor tyrosine kinases (RTK) and integrins co-controls cancer cell survival and therapy resistance. However, co-dependencies between these receptors and therapeutically exploitable vulnerabilities remain largely elusive in HPV-negative head and neck squamous cell carcinoma (HNSCC).

**Methods:**

The cytotoxic and radiochemosensitizing potential of targeting 10 RTK and β1 integrin was determined in up to 20 3D matrix-grown HNSCC cell models followed by drug screening and patient-derived organoid validation. RNA sequencing and protein-based biochemical assays were performed for molecular characterization. Bioinformatically identified transcriptomic signatures were applied to patient cohorts.

**Results:**

Fibroblast growth factor receptor (FGFR 1–4) targeting exhibited the strongest cytotoxic and radiosensitizing effects as monotherapy and combined with β1 integrin inhibition, exceeding the efficacy of the other RTK studied. Pharmacological pan-FGFR inhibition elicited responses ranging from cytotoxicity/radiochemosensitization to resistance/radiation protection. RNA sequence analysis revealed a mesenchymal-to-epithelial transition (MET) in sensitive cell models, whereas resistant cell models exhibited a partial epithelial-to-mesenchymal transition (EMT). Accordingly, inhibition of EMT-associated kinases such as EGFR caused reduced adaptive resistance and enhanced (radio)sensitization to FGFR inhibition cell model- and organoid-dependently. Transferring the EMT-associated transcriptomic profiles to HNSCC patient cohorts not only demonstrated their prognostic value but also provided a conclusive validation of the presence of EGFR-related vulnerabilities that can be strategically exploited for therapeutic interventions.

**Conclusions:**

This study demonstrates that pan-FGFR inhibition elicits a beneficial radiochemosensitizing and a detrimental radioprotective potential in HNSCC cell models. Adaptive EMT-associated resistance appears to be of clinical importance, and we provide effective molecular approaches to exploit this therapeutically.

**Supplementary Information:**

The online version contains supplementary material available at 10.1186/s12943-024-01954-8.

## Background

Head and neck squamous cell carcinoma (HNSCC) is a cancer type of unmet need and encompasses a heterogeneous group of tumors with a generally poor prognosis, especially in the population of human papillomavirus (HPV)-negative HNSCC [1]. Owing to the fact that the 5-year overall survival (OS) ranges around 50% upon conventional radio(chemo)therapy, novel, particularly molecular-targeted approaches are warranted [2]. Such therapies require a detailed understanding of molecular vulnerabilities of HNSCC, which are based on genetic and epigenetic alterations as well as microenvironmental factors like growth factors, extracellular matrix and their cognate receptors [3, 4]. Although receptor tyrosine kinases (RTK) commonly drive HNSCC progression, the majority of targeted approaches against them demonstrated non-beneficial response rates regarding improvement in locoregional control, overall survival and quality of life [1, 2].

More recently, large multi-omics profiling with higher resolution redefined the molecular landscape of HNSCC, potentially driving the discovery of new biomarkers and molecular targets [5, 6]. Examples of emerging RTK are cMET [6, 7], AXL [6, 7], or the fibroblast growth factor receptor family (FGFR1-4) [6, 8]. FGFR are frequently overexpressed in HPV-negative tumors and are considered as strong determinants of prognosis and resistance to radio(chemo)therapy. Hyperactivated FGFR forms are thought to trigger epithelial-to-mesenchymal transition (EMT) in cancer [9]. EMT, which is highly conserved and tightly regulated during development, is an inevitable feature of malignancy in which polarized epithelial cells acquire mesenchymal properties like local invasion, metastatic spread and therapeutic resistance [10]. Based on reports of FGFR overexpression and EMT presence in HNSCC, it is intriguing that studies elucidating the interplay of FGFR and EMT for HNSCC are extremely scarce. Clarifying this connection offers the potential for both a better mechanistic understanding and the identification of new, potent target molecules for therapy optimization.

RTK and integrins, the largest family of transmembrane cell adhesion molecules, coalesce at certain junctions of the cell membrane, the so-called focal adhesions. Mutual and cooperative interactions of these transmembrane receptors co-regulate not only EMT but also other cell functions such as survival, proliferation and migration/invasion [11, 12]. How essential these interdependencies are is evident for a number of preclinical studies reporting enhanced tumor cell kill and radiochemosensitization when the epidermal growth factor receptor (EGFR) is inhibited simultaneously to β1 integrin [4, 13]. Interestingly, 20% of investigated HNSCC cell models were refractory to this dual inhibition, putatively representing a particular resistant HNSCC patient subcohort.

We here hypothesized that similar exploitable therapeutic vulnerabilities exist for other RTK/β1 integrin combinations. To address this issue, we selected ten RTK with existing FDA-approved drugs together with β1 integrin for an RNAi-based screen in which FGFR1—4 showed the strongest cell killing and radiochemosensitizing-potential independent from β1 integrin. In a heterogeneous panel of 20 HNSCC cell models cultured under 3D laminin-rich extracellular matrix (lrECM) conditions, we corroborated this observation and distinguished a responsive and unresponsive subgroup. By focusing on the newly identified FGFR inhibitor (FGFRi)-induced adaptive resistance/radioprotection, RNA-sequencing (RNA-seq) revealed distinct EMT properties associated with the opposing responses to FGFRi. The synergy of bioinformatics and functional kinase inhibitor screening facilitated the identification of drug targets whose inhibition could be exploited to overcome FGFRi-induced adaptive resistance in cell models and HNSCC organoids alike. Finally, the translation of the EMT-associated transcriptomic profile to HNSCC patient cohorts showed prognostic value and confirmation of therapeutically exploitable EGFR-related vulnerabilities.

## Results

**Fig. 1:**
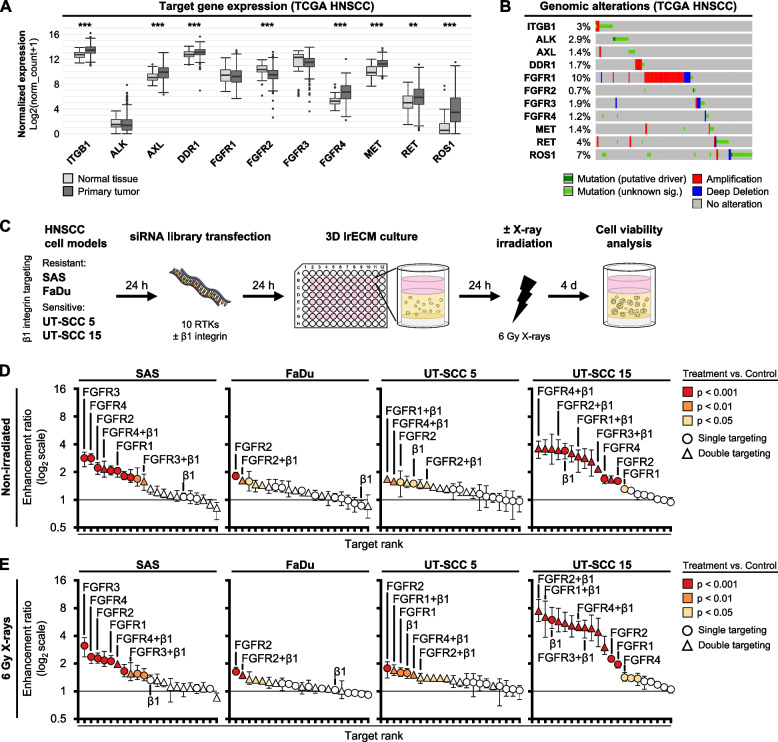
RNAi cell viability screen identifies FGF-receptors as potent cytotoxic and radiosensitizing targets for HNSCC cell models with and without simultaneous β1 integrin targeting. **A** Gene expression analysis of 10 selected, clinically relevant RTK with available FDA-approved drugs (Table S2) in HPV-negative HNSCC patients of the TCGA cohort. Data of normalized primary tumor (*n* = 415) and normal tissues (*n* = 44) were compared using unpaired t test (***p ≤ 0.001, **p ≤ 0.01). **B** OncoPrint of target gene alterations in HPV-negative HNSCC patients of the TCGA cohort (*n* = 415). Percentage indicates the proportion of patients with genetic alterations. Only data of patients with alterations are shown. **C** Workflow of RNAi cell viability screen in 3D lrECM (laminin-rich extracellular matrix) HNSCC cell models. Images were partly adapted from Servier Medical Art by Servier, licensed under a Creative Commons Attribution 3.0 Unported License. **D** Enhancement ratios (ER) of cell viability of 3D lrECM HNSCC models upon single or double siRNA-mediated knockdowns of 10 RTK and β1 integrin (labeled as β1). ER and statistics are derived from corresponding cell viability data (Fig. S2A) and presented as mean ± range (*n* = 3; two-way ANOVA; Dunnett’s multiple comparison test to corresponding controls). **E** ER of cell viability of 6 Gy X-ray irradiated 3D lrECM HNSCC models upon single or double knockdowns of 10 RTK and β1 integrin. ER and statistics are derived from the corresponding cell viability data (Fig. S2B) and presented as mean ± range (*n* = 3; two-way ANOVA; Dunnett’s multiple comparison test to corresponding irradiated controls)

We commenced our study by RNAi-based targeting of selected RTK with available FDA-approved drugs (Table S2). All ten receptor candidates are underexplored in HNSCC, and the majority exhibit oncogenic alterations in patients (Fig. 1A-C). In four exome-characterized HNSCC cell models (Fig. S1A-D), we detected cell model-dependent enhancement ratios (ER) for the various single and simultaneous knockdown combinations ranging between an ER of 0.9 (no effect on survival) to an ER of 7.5 (high cytotoxic/radiosensitizing effect) (Fig. 1D-E; Fig. S2A-B). Targeting of the fibroblast growth factor receptor family members (FGFR1-4), especially FGFR1 and FGFR2, mediated significant reduction of basal cell survival and radiosensitization relative to other RTK and respective controls (Fig. 1D-E; Fig. S1E). The simultaneous depletion of RTK and β1 integrin showed mainly overlapping or even reduced effectiveness compared with single knockdowns. Only in UT-SCC 5 and UT-SCC 15 cell models, certain β1 integrin/FGFR knockdown combinations resulted in minimal enhancement (Fig. 1D-E). Overall, our RNAi cell viability screen identified the four expressed members of the FGFR family (FGFR1-4; Fig. S1F) as the most promising RTK candidates, whose depletion enhanced cytotoxicity and cellular radiosensitivity. Furthermore, these data indicate a more profound impact of RTK than β1 integrin on cell viability per se and after genotoxic stress such as irradiation in the 3D lrECM HNSCC cell models.

### Pharmacological inhibition of FGFR and β1 integrin elicits a sensitization-to-protection response pattern in 3D HNSCC cell models

**Fig. 2:**
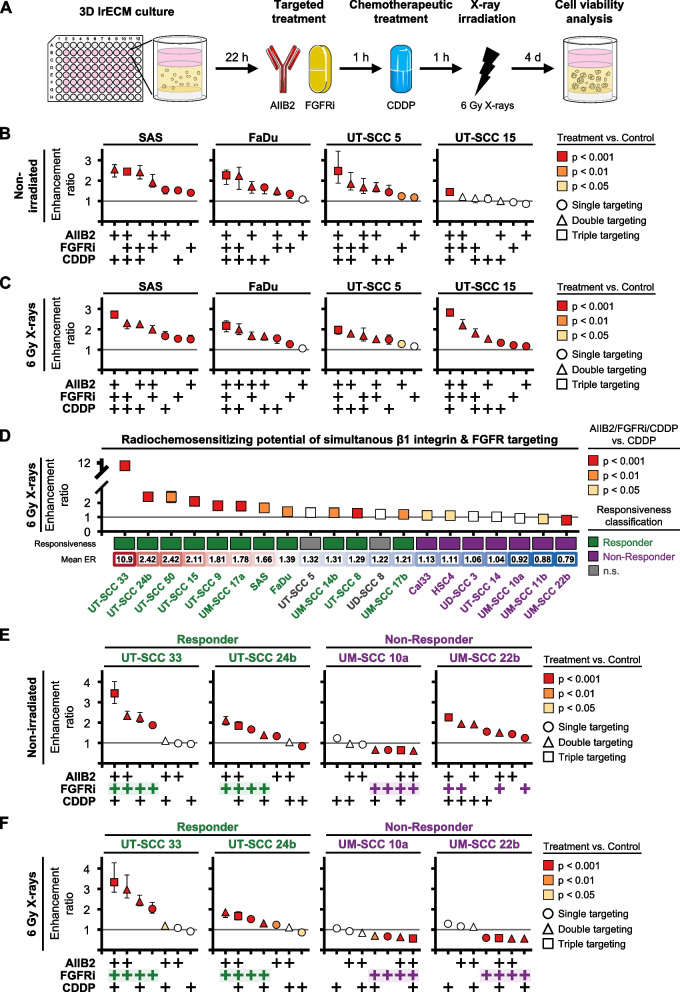
Pharmacological FGFR/β1 integrin inhibition plus Cisplatin induces a broad, sensitizing-to-resistant viability spectrum in irradiated 3D HNSCC cell models. **A** Workflow of drug cell viability screen in 3D lrECM HNSCC cell models upon treatment using anti-β1-integrin mAb (AIIB2; 20 µg/ml), pan-FGFR inhibitor (Erdafitinib, FGFRi; 2 µM) and Cisplatin (CDDP; 0.5 µM) with or without single 6 Gy X-ray irradiation. Images were partly adapted from Servier Medical Art by Servier, licensed under a Creative Commons Attribution 3.0 Unported License. **B** Enhancement ratios (ER) of cell viability responses of indicated cell models to single, double and triple applications of AIIB2, FGFRi and CDDP. DMSO/IgG were used as controls. ER and statistics are derived from the corresponding cell viability data (Fig. S3A) and presented as mean ± range (*n* = 3, two-way ANOVA, Dunnett’s multiple comparison test to corresponding controls). **C** ER of cell viability responses of indicated 6 Gy X-ray irradiated cell models to single, double and triple applications of AIIB2, FGFRi and CDDP. DMSO/IgG were used as control. ER and statistics are derived from the corresponding cell viability data (Fig. S3B) and presented as mean ± range (*n* = 3; two-way ANOVA; Dunnett’s multiple comparison test to corresponding irradiated controls). **D** ER of cell viability responses of 20 indicated cell models comparing the triple combination AIIB2, FGFRi and CDDP to the corresponding single CDDP treatments with a single dose of 6 Gy X-ray irradiation. The adapted ER (AIIB2/FGFRi/CDDP vs. CDDP) and statistics are derived from corresponding cell viability data (Fig. S3D) and presented as mean ± range (Two-way ANOVA; Tukey multiple comparison test; ***p ≤ 0.001, **p ≤ 0.01, *p ≤ 0.05, n.s. *p* > 0.05). **E** ER of cell viability responses of indicated cell models to the single, double or triple combination with AIIB2, FGFRi and CDDP. DMSO/IgG were used as control. ER and statistics are derived from the corresponding cell viability data (Fig. S4A) and presented as mean ± range (*n* = 3; two-way ANOVA; Dunnett’s multiple comparison test to corresponding controls). **F** ER of cell viability responses of indicated cell models to the single, double or triple combination of AIIB2, FGFRi and CDDP plus 6 Gy X-ray irradiation. DMSO/IgG were used as control. ER and statistics are derived from the corresponding cell viability data (Fig. S4B) and presented as mean ± range (*n* = 3; two-way ANOVA; Dunnett’s multiple comparison test to corresponding irradiated controls)

Considering the RNAi-mediated knockdown dynamics and the fact that molecularly targeted drugs rather than RNAi technology are used clinically, we chose a more translational approach with the pan-FGFR small molecule inhibitor (SMI) Erdafitinib (FGFRi), the β1-integrin-antagonizing monoclonal antibody AIIB2 and the standard chemotherapy drug cisplatin (CDDP) with and without a single dose of 6 Gy X-ray irradiation (Fig. 2A). Simultaneous FGFRi/AIIB2 application was superior to mono-targeting and markedly declined cell viability and enhanced both chemo- and radiosensitivity in a cell model-dependent manner (Fig. 2B-C, Fig. S3A-B). With the addition of CDDP, all four HNSCC cell models showed the highest levels of cytotoxicity and radiosensitization despite their heterogeneous susceptibility to single and double drug exposures (Fig. 2B-C, Fig. S3A-B).

Next, we expanded our investigation on the radiochemosensitizing potential of FGFRi/AIIB2/CDDP treatment to 16 additional HPV-negative HNSCC cell models. Significant ERs ranging from 1.17 to 7.9 were consistently observed for the triple treatment compared to CDDP (Fig. S3C). In combination with irradiation, however, cell model-dependent vulnerabilities emerged, which divided the cell model panel into responder and non-responder groups (Fig. 2D, Fig. S3D). We based this distinction on the significant benefit of the triple treatment over CDDP with an adapted ER of ≥ 1.2 or the absence of effectiveness at ER levels < 1.2. Our interest was sparked by the strong resistance of certain cell models towards our multi-targeting approach.

Hence, we selected the non-responding UM-SCC 10a and UM-SCC 22b as well as the responding UT-SCC 33 and UT-SCC 24b cell models for subsequent investigations. In the responders, we confirmed that the combinations of AIIB2/FGFRi and AIIB2/FGFRi/CDDP with and without irradiation produced the highest level of cell kill (Fig. 2E-F). However, in the non-responders, marked differences were evident in the basal, but not in the radiation response to AIIB2, FGFRi and CDDP exposure (Fig. 2E-F, Fig. S4A-B). While UM-SCC 22b cells showed an ER of approximately 2.2 upon triple treatment, they were almost unresponsive to single FGFRi (Fig. 2E-F, Fig. S4A-B). In contrast, UM-SCC 10a cells remained in their FGFRi-resistant state regardless of all other drug combinations and radiation applied (Fig. 2E-F, Fig. S4A-B). This result prompted us to decipher the responsiveness to FGFRi monotherapy in more detail, unravel the underlying mechanisms and investigate their therapeutic exploitability.

### The cyto- and radioprotective effect induced by FGFR inhibition is concentration- and FGFR3-dependent

**Fig. 3:**
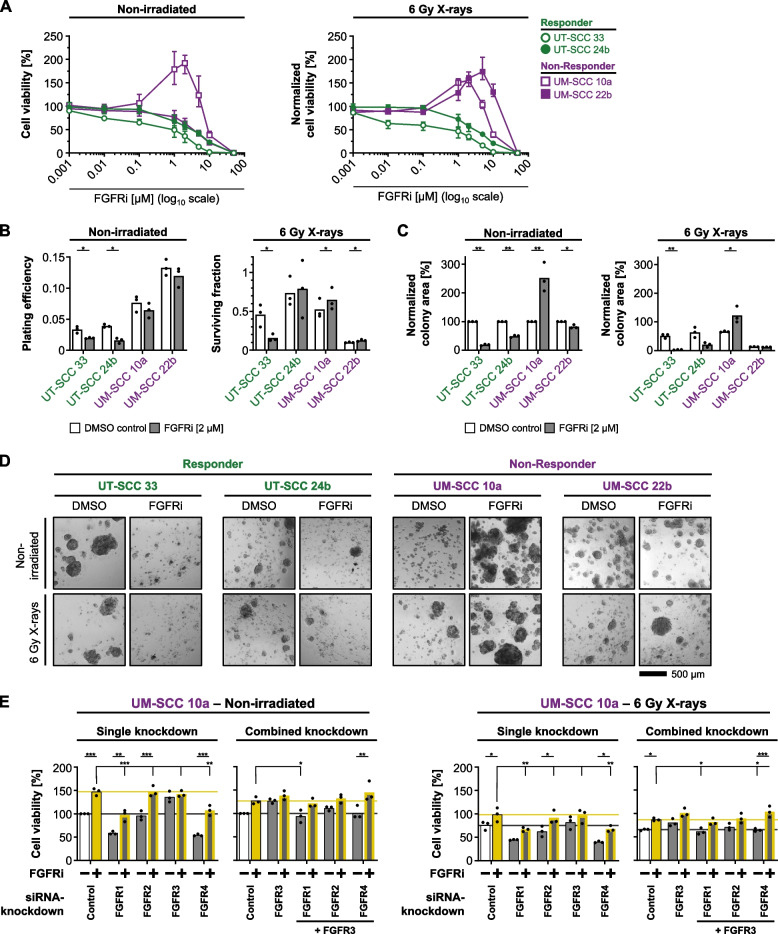
Pharmacological FGFR inhibition elicits Janus-faced response patterns in non-irradiated and irradiated 3D lrECM HNSCC models. **A** Mean cell viability (± range) of cell models to indicated FGFRi concentrations (*n* = 3) at non-irradiated (left panel) or 6 Gy X-ray irradiated (right panel) conditions. Normalization was performed to corresponding non-irradiated/irradiated controls. **B** Plating efficiencies and surviving fractions of indicated cell models treated with FGFRi with or without a single dose of 6 Gy X-rays based on the colony number counts (*n* = 3; mean ± range; paired t-test; *p ≤ 0.05). **C** Normalized colony area of indicated cell models treated with FGFRi. The absolute area was normalized to the mean of the corresponding non-irradiated control (*n* = 3; mean ± range; paired t-test; **p ≤ 0.01, *p ≤ 0.05). **D** Representative focus stacked bright-field images of colony formation assays of 3D lrECM HNSCC cell models exposed to FGFRi (DMSO used as control) plus/minus 6 Gy X-ray irradiation. **E** Cell viability of 3D lrECM grown UM-SCC 10a cells upon single or double siRNA-mediated knockdowns of indicated genes under non-irradiated and 6 Gy X-ray irradiated conditions (*n* = 3; mean; two-way ANOVA; Tukey multiple comparison test; ***p ≤ 0.001, **p ≤ 0.01, *p ≤ 0.05). Non-targeting siRNAs were used as control

We commenced these analyzes by covering FGFRi concentrations between 0.001 and 50 µM and found a concentration-dependent, wave-like pattern of cytoprotection and radioprotection induced by FGFRi in FGFR-expressing UM-SCC 10 cells, while UM-SCC 22b cells only showed radioprotection (Fig. 3A). FGFR-expressing responder models showed, in opposition, a concentration-dependent decline in cell survival (Fig. 3A). These findings were corroborated by colony formation data demonstrating FGFRi-induced radiosensitization in UT-SCC 33 cells, while both non-responders exhibited elevated surviving fractions with highly increased colony areas in UM-SCC 10a cells (Fig. 3B-D). To exclude off-target effects and explore mechanistic insights, we selected the strongest non-responding cell model, UM-SCC 10a, for siRNA-based single and combinatory FGFR1-4 knockdowns. Intriguingly and in contrast to FGFR1 and FGFR4, which reduced cell viability significantly, we observed a similar increase in cell viability upon FGFR3 depletion as for FGFRi treatment (Fig. 3E; left panels). However, the application of FGFRi also significantly increased cell viability under the knockdown of FGFR1, 2 and 4. Since FGFR3 seems to play a central role, we combined FGFR3 silencing with silencing of FGFR1, 2 or 4 (Fig. 3E). Interestingly, knockdown of FGFR3 was able to reduce the cytotoxic knockdown effects of FGFR1 and 4, which then presented superimposable to those observed for knockdown of FGFR 1 or 4 combined with FGFRi (Fig. 3E). A similar response pattern to the various FGFR depletions plus/minus FGFRi were detectable when X-ray irradiation was added (Fig. 3E; right panels). These data suggest two key aspects: (i) FGFR3 can be considered central to the cyto- and radioprotective effects of the pharmacological FGFR inhibition; (ii) yet to be identified survival-promoting bypass signaling pathways appear to be induced by FGFRi during depletion of FGFR1, 2 and 4. These findings prompted us to address the underlying adaptation mechanisms through RNA sequencing.

### FGFRi responding and non-responding HNSCC cell models demonstrate profound differences in their transcriptomic landscape

**Fig. 4:**
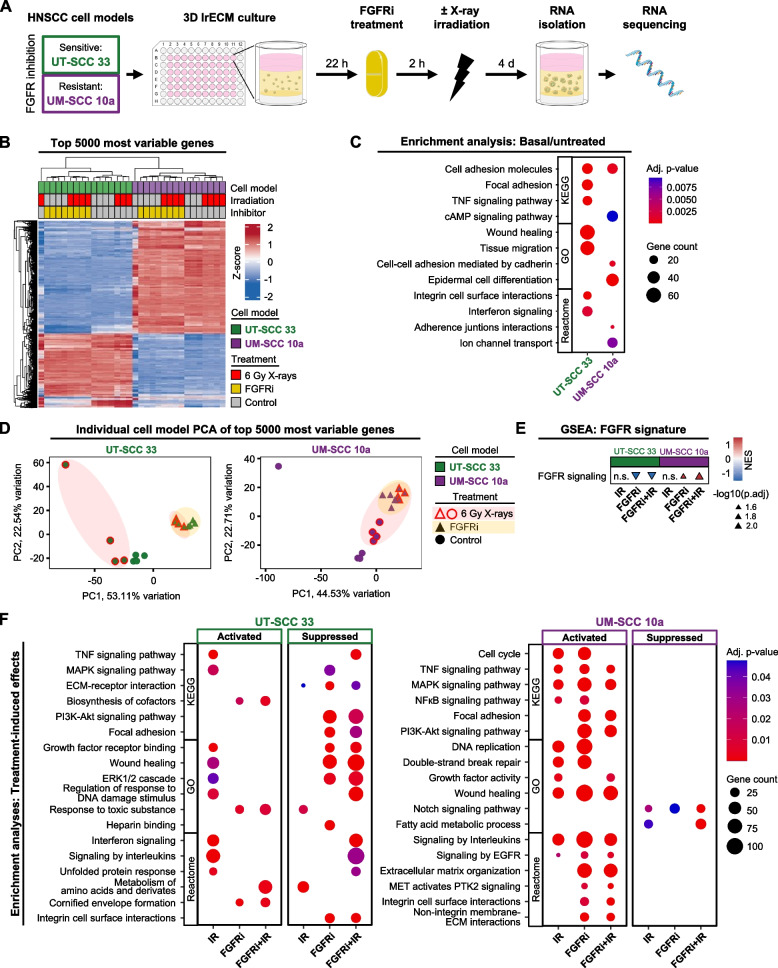
The transcriptomic landscape of FGFRi sensitive and resistant HNSCC cell models differs profoundly. **A** Workflow of RNA-sequencing analysis in 3D lrECM HNSCC cell models upon FGFRi treatment (2 µM; DMSO as control) with or without single 6 Gy X-ray irradiation. Images were partly adapted from Servier Medical Art by Servier, licensed under a Creative Commons Attribution 3.0 Unported License. **B** Heatmap of 5000 most variable expressed genes between all treated and control samples. Columns represent biological replicates (*n* = 4), rows represent z-score normalized gene expression data, both hierarchically clustered. **C** Overrepresentation analyses of strongly differential expressed genes (DEG, log2FC ≥|2|) between UT-SCC 33 and UM-SCC 10a cell models at basal/untreated conditions. Selected functional enrichments of individual database analyses (KEGG, GO, Reactome) are presented by gene counts and adjusted *p*-values. Complete results are listed in Table S3. **D** Principal component analysis of the top 5000 most variably expressed genes in each cell model upon indicated treatment conditions. Colored ellipses outline directions of treatment-induced transcriptomic shifts compared to controls. **E** Normalized enrichment score (NES) summary of six gene set enrichment analyses (GSEA) for the FGFR signaling signature (Table S3) in each DEG comparison group (IR, 6 Gy X-ray irradiated; FGFRi, FGFR inhibitor treatment; FGFRi/IR, combined treatment). Significance levels (p.adj, adjusted *p*-value ≤ 0.05; n.s., non-significant) are indicated by triangle size. Triangle direction represents enrichment or suppression in the corresponding DEG comparison group. **F** Functional characterization of treatment-induced effects between the DEG comparison groups (IR; FGFRi; FGFRi/IR) for each cell model. All DEG (adjusted *p*-value ≤ 0.05) were included. Selected functional enrichments of individual database analyses (KEGG, GO, Reactome) are presented by gene counts and adjusted *p*-values. Complete results are listed in Table S3

A comparative characterization of the transcriptomic response (Fig. 4A) to FGFRi/irradiation in UT-SCC 33 and UM-SCC 10 cells already demonstrated strong differences in basal/untreated conditions (Fig. 4B-C, Fig. S5A-B). We observed 7596 and 9902 overexpressed genes (coding and non-coding) in sensitive and resistant cell models, respectively (adj. *p*-value ≤ 0.05; Fig. S5B-C). Functional profiling on multiple pathway databases through enrichment of highly differentially expressed genes (DEG, log2 fold-change ≥ 2) uncovered different sets of cell adhesion and ECM-organizing molecules upregulated in both cell models (Fig. 4C, Table S3). Sensitive UT-SCC 33 cells showed strong enrichment for migration, wound healing, focal adhesion signaling including integrin cell surface interactions. In contrast, an overrepresentation of cadherin cell–cell interactions in combination with an enrichment for transport channels was exhibited in resistant UM-SCC 10a cells (Fig. 4C).

To connect transcriptomic changes to treatment response, we next performed a principal component analysis. It outlines an allocation of the transcriptomic profiles from sensitive irradiated and irradiated/FGFRi-treated UT-SCC 33 cells into separable groups (Fig. 4D; marked with colored circles). In opposition, resistant UM-SCC 10a cells show an overlapping allocation of their profiles of these two treatment groups (Fig. 4D). We then determined the DEG (adj. *p*-value ≤ 0.05) in the three treatment-to-control pairs for each cell model (Fig. S5C-D) and tested for general treatment-induced changes in FGFR signaling by gene set enrichment analysis (GSEA) (Fig. 4E). We found that FGFRi mediated a highly significant downregulation of FGFR signaling in the sensitive UT-SCC 33 and an enhanced FGFR signaling in the resistant UM-SCC 10a cells (Fig. 4E). Moreover, irradiation alone caused an activation of TNF, MAPK, DNA damage stimuli and interleukin-related signaling among others, while suppression was only marginally (Fig. 4F). FGFRi alone or combined with irradiation, however, accomplished a broad suppression of prosurvival signaling via PI3K/Akt, focal adhesion and growth factor receptors among others in the sensitive UT-SCC 33 cells (Fig. 4F). While irradiation alone caused comparable responses in resistant UM-SCC 10a cells, single FGFRi and FGFRi/irradiation elicited profound activations ranging from an extensive cancer adhesome restructuring to signaling cascades associated with MAPK, EGFR or PI3K/Akt (Fig. 4F). Taken together, our findings provide evidence for differential transcriptomic response patterns towards FGFR inhibition.

### FGFRi treatment differentially regulates the EMT profile in sensitive and resistant HNSCC cell models

**Fig. 5:**
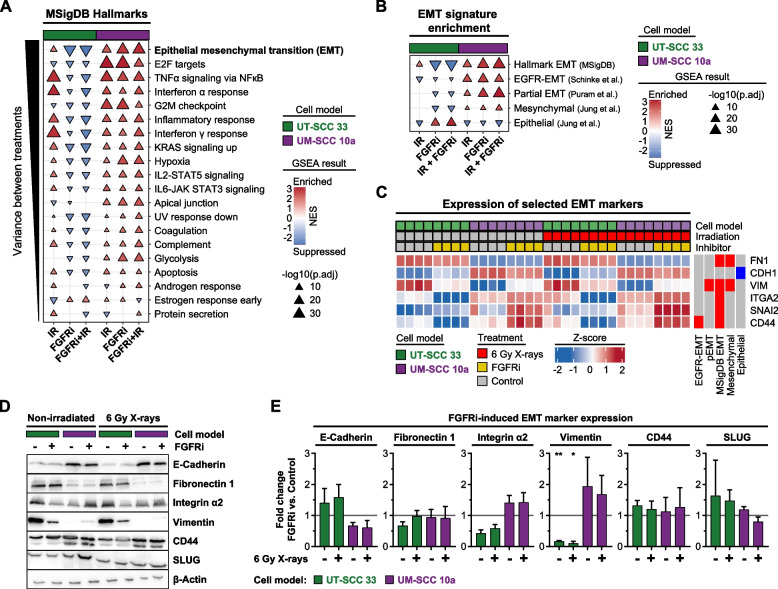
The EMT profile is altered most strongly and in opposite directions in sensitive versus resistant HNSCC cell models after FGFR inhibition. **A** Normalized enrichment score (NES) summary of six gene set enrichment analyses (GSEA) for MSigDB hallmark gene sets. Top 20 hallmarks with the highest variance between DEG comparison groups (*n* = 4; IR, 6 Gy X-ray irradiated; FGFRi, FGFR inhibitor treatment; FGFRi/IR, combined treatment) are depicted. Significance levels (p.adj, adjusted *p*-value ≤ 0.05) are indicated by triangle size. Triangle direction represents enrichment or suppression in the corresponding DEG comparison group per cell model. **B** NES summary graph of multiple GSEA of indicated HNSCC-related EMT gene sets (Table S3) in each DEG comparison group of both cell models. Significance levels (adjusted *p*-value ≤ 0.05) are indicated by triangle size. Triangle direction represents enrichment or suppression in the corresponding DEG comparison group. **C** Expression heatmap of selected EMT marker genes in both cell models. Genes are annotated by their corresponding HNSCC gene signatures (Table S3). Columns represent individual biological replicates; rows are clustered hierarchically. **D** Western blot analysis of selected EMT marker proteins from whole cell lysates of 3D lrECM grown cell models upon indicated treatments. β-actin served as loading control. Representative blots are shown. Where indicated, cells were treated with 2 µM FGFRi (DMSO was used as control). **E** Densitometric analyses of EMT marker expression shown in ‘D’. Mean fold changes (± standard deviation;* n* = 3) compared to corresponding non-irradiated/irradiated controls are shown. All samples were normalized to their corresponding β-actin loading control (Two-way ANOVA utilizing normalized densitometry data, Tukey multiple comparison test, **p ≤ 0.01; *p ≤ 0.05)

To uncover biomarkers and therapeutically exploitable HNSCC vulnerabilities associated with the differential transcriptomic response patterns, we utilized the MSigDB hallmark gene sets. Comparative GSEA revealed mesenchymal characteristics for UT-SCC 33 cells and epithelial for UM-SCC 10a cells (Fig. S6A-B). The hallmark of EMT was most profoundly affected in all DEG group comparisons upon treatment (Fig. 5A-B). While both cell models displayed great similarities in activated EMT signatures upon irradiation, it was FGFRi application that simultaneously induced and catalyzed the opposing response patterns. Upon FGFRi exposure, EMT traits were strongly upregulated in resistant UM-SCC 10a and maximally downregulated in UT-SCC 33 cells (Fig. 5A); a finding corroborated in independent HNSCC-specific gene sets for various EMT-states (Fig. 5B). These results suggest that FGFR inhibition reverses the mesenchymal phenotype of the sensitive HNSCC cell model and induces it in the resistant one.

We subsequently validated our RNA-seq datasets on protein level of selected, contrary regulated EMT markers in both cell models (Fig. 5D-E). We confirmed the basal phenotype as well as most EMT marker deregulations upon FGFRi and irradiation. Notably were the simultaneous upregulation of mesenchymal markers and stable epithelial marker expression during FGFRi resistance response in UM-SCC 10a cells. Corroborated by the upregulation of the early EMT transcription factor SLUG, this points towards a switch into a partial EMT (pEMT) phenotype as a basis of this profound and highly proliferative resistance mechanism (Fig. S6C).

### Targeting of upregulated, EMT-related kinases diminishes resistance in UM-SCC 10a cells

**Fig. 6:**
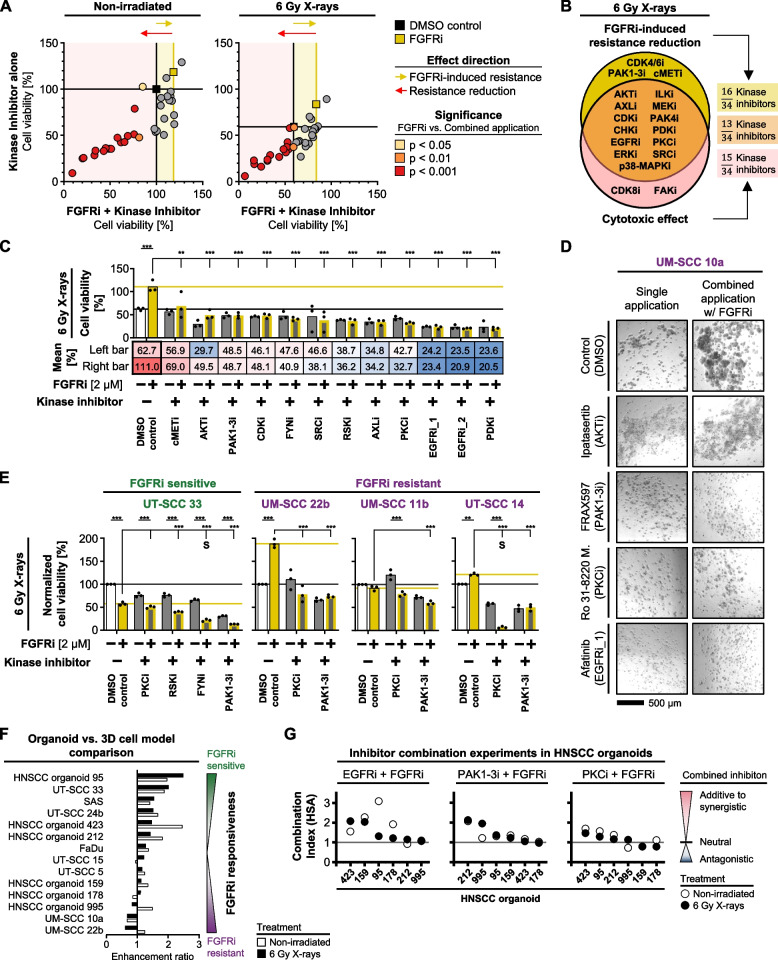
Pharmacological inhibition of specific EMT-associated kinases reduces FGFRi-induced resistance. **A** Drug cell viability screen in non-irradiated (left panel) and irradiated (right panel) UM-SCC 10a cells upon monotherapy with selected kinase inhibitors alone (y-axis) versus dual therapy with selected kinase inhibitors plus FGFRi (x-axis). Corresponding annotated cell viability data are presented in Fig. S8 (*n* = 3; two-way ANOVA; Dunnett’s multiple comparison test to corresponding single FGFRi treatment). **B** Venn diagram of significant kinase inhibitor effectiveness shown in Fig. 6A (right panel). Underlying data and statistics are presented in Fig. S8. **C** Effects on cell viabilities of irradiated UM-SCC 10a cells upon exposure to concentration-optimized kinase inhibitors listed in Fig. 6B. The inhibitors RSKi and EGFR_2 were added to the panel (inhibitor names, cell viability data of non-irradiated cells and applied concentrations are displayed in Fig. S9A). Bars and bottom annotation table display mean cell viability (*n* = 3; two-way ANOVA; Tukey multiple comparison test; ***p ≤ 0.001, **p ≤ 0.01, *p ≤ 0.05). **D** Representative focus-stacked images of colony formation of 3D lrECM UM-SCC 10a cell cultures upon indicated treatments. Quantitative analysis is presented in Fig. S9B-C. **E** Comparative testing of cell viability in one FGFRi sensitive versus three FGFRi resistant 3D HNSCC cell models upon indicated mono- and combination treatments relative to corresponding controls (inhibitor names, cell viability data of non-irradiated cells and applied concentrations are displayed in Fig. S10A; cell viability data of irradiated cells normalized to non-irradiated controls are shown in Fig. S10B). Bars represent mean cell viability (*n* = 3; two-way ANOVA; Tukey multiple comparison test; ***p ≤ 0.001, **p ≤ 0.01). ‘S’ indicates synergy calculated by the Bliss independence model. **F** Comparison of FGFRi responsiveness in 3D lrECM cell models and HNSCC organoids in absence and presence of 6 Gy X-rays. Bars represent mean enhancement ratio of three biological replicates per cell model (2 µM FGFRi) and six technical replicates per organoid (1.5 µM FGFRi). Corresponding cell viability data are listed in Fig. S3A-B and Fig. S4A-B for cell models and Fig. S11B-C for HNSCC organoids. **G** Combinatory effectiveness plots of three kinase inhibitors (EGFRi, PAK1-3i, PKCi) together with FGFRi in indicated HNSCC organoids. Results are presented according to the highest-single agent (HSA) combination index, where scores > 1 indicate a potential additive to synergistic effect. Corresponding cell viability data are shown in Fig. S11B-C

Next, we hypothesized that these differential EMT-related, transcriptomic profiles can be therapeutically exploited and selected a subset of either the generally enriched or FGFRi-induced druggable kinases (Fig. S7A-C). Among the 34 chosen small molecule inhibitors (Fig. 6A), two induced cell kill without affecting FGFRi-induced radioprotection, three reduced FGFRi-mediated resistance without cytotoxicity, and 13 showed both cytotoxicity as monotherapy and antagonization of the FGFRi-induced resistance (Fig. 6A-B, Fig. S8). Additional testing with adjusted inhibitor concentrations for cell viability and colony area revealed a potential drug list with resistance-reducing efficacy including inhibitors for PKC, RSK, FYN and PAK1-3 among others (Fig. 6C-D, Fig. S9A-C). We comparatively evaluated these four kinase inhibitors, namely Ro 31–8220 Mesylate (PKCi), BI-D1870 (RSKi), PP2 (FYNi), FRAX597 (PAK1-3i), in the FGFRi-sensitive cell model UT-SCC 33 and the FGFRi-resistant cell models UM-SCC 11b, UT-SCC 14, UM-SCC 22b. Interestingly, single and concomitant use of these inhibitors together with FGFRi elicited cytotoxic and radiosensitizing effects to varying degrees in both sensitive UT-SCC 33 and resistant UM-SCC 11b, UT-SCC 14, and UM-SCC 22b cell models (Fig. 6E, Fig. S10).

To test our results in a model that is even closer to the clinic, we selected six HNSCC organoids that cover the full spectrum of FGFRi responsiveness, similar to the cell models we used (Fig. 6F, Fig. S11A). Single and combinatory drug applications were performed with PKCi, PAK1-3i and the FDA-approved EGFRi Lapatinib in absence and presence of X-ray irradiation. The EGFRi/FGFRi combination had a radiosensitizing and even partly synergistic effect in half of the organoids (Fig. 6G, Fig. S11B). Interestingly, the most unresponsive organoids to this combination (#212, #995) displayed a sensitivity for PAK1-3i/FGFRi after irradiation. Overall, our results show that the adaptive response triggered by FGFR inhibition involves dependencies on different signaling circuits that may also contribute to a radioprotective response. These dependencies appear to be identifiable vulnerabilities in HNSCC for therapeutic exploitation.

### EGFR-related gene signatures connect FGFRi-elicited resistance to clinical HNSCC cohorts

**Fig. 7:**
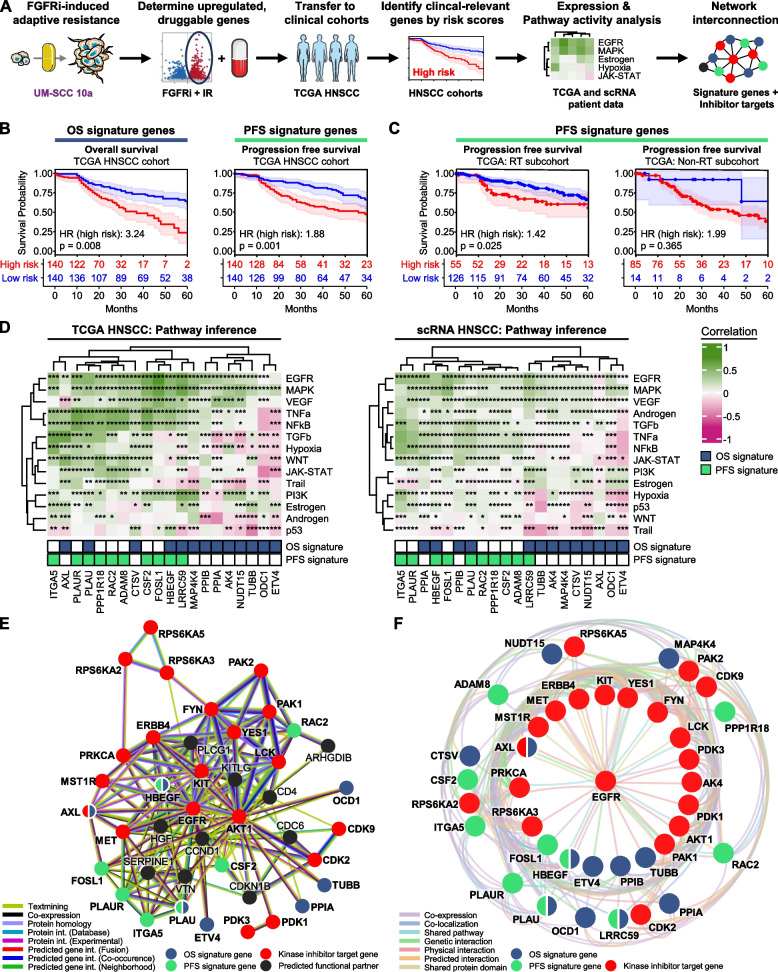
In vitro resistance signatures predict clinical outcome and clinically relevant target genes in HNSCC patient cohorts. **A** Workflow of defining clinical outcome and clinically relevant targets identified in the FGFRi-induced adaptive resistance response in UM-SCC 10a cells. Images were partly adapted from Servier Medical Art by Servier, licensed under a Creative Commons Attribution 3.0 Unported License. **B** Stratification of HPV-negative HNSCC patients from the training cohort (TCGA,* n* = 280 patients with available clinical endpoints and target gene expression) with the indicated signature-based risk scores (median cut-off) for overall (OS) and progression-free survival (PFS). Hazard ratios of high-risk patients (red curves) and log-rank test *p*-values for the comparison of high- and low-risk groups are indicated together with 95% confidence intervals in Kaplan–Meier curves including patient numbers at risk. Signature genes and coefficients are listed in Table S3. **C** Radiotherapy-treated (RT) and non-RT subcohorts of the HPV-negative HNSCC training cohort (RT, *n* = 181 patients; non-RT, *n* = 99 patients) are stratified with the PFS signature-based risk score (median cut-off) for PFS. Hazard ratios of high-risk patients (red curves) and log-rank test *p*-values for the comparison of high- and low-risk groups are indicated together with 95% confidence intervals in Kaplan–Meier curves including patient numbers at risk. **D** Spearman correlations of derived pathway activities with the corresponding expression of OS/PFS signature genes in either HPV-negative HNSCC TCGA patients (left; *n* = 415) or single HNSCC cells (right; *n* = 1891 cells from *n* = 10 patients; GSE103322; scRNA, single cell RNA-sequencing). Correlations are hierarchically clustered and annotated with adjusted *p*-values for correlation significance (***p ≤ 0.001, **p ≤ 0.01, *p ≤ 0.05). **E** Interaction network of OS and PFS signature genes and kinase inhibitor targets. STRING database was used to discover interconnections between the targets of identified resistance-overcoming kinase inhibitors (see Fig. 6C) and predicted functional partners. The evidence color key for protein- and gene-level connections is indicated. **F** Interaction network of OS and PFS signature genes and kinase inhibitor targets. GeneMania database was applied to uncover interconnections between the targets of identified resistance-overcoming kinase inhibitors (see Fig. 6C) in radial layout. Evidence color key for pathway, protein- and gene-level connections is shown

Subsequently, we addressed whether the identified resistance signatures and their druggability observed in multiple cell models grown under more physiological 3D, matrix-based cell culture conditions are clinically detectable and applicable in patients with HPV-negative HNSCC from the TCGA cohort. Intriguingly, upon defining the set of druggable DEG upregulated during the FGFRi-induced radioprotective response of UM-SCC 10a cells (Fig. 7A, Table S3), we identified a 13-gene signature for overall survival (OS) (Fig. S12A) and a 10-gene signature for progression-free survival (PFS) (Fig. S12B) by forward-feature selection and multivariate Cox regression models. The generation of prognostic risk scores for OS and PFS successfully stratified patients into high- and low-risk groups (median threshold). For each endpoint, the high-risk groups correlated significantly with reduced survival in the TCGA training cohort (Fig. 7B) as well as in two HNSCC validation cohorts from Fred Hutchinson and MD Anderson Cancer Centers (Fig. S12C; FHCRC, *n* = 59; MDACC-HNSCC, *n* = 73). In addition, the 10-gene signature prognosticated PFS in patients treated with radiotherapy (RT), but not in patients from the TCGA cohort who were not treated with RT (Fig. 7C).

Unifying the OS- and PFS-derived signatures resulted in a total of 20 unique genes with potential druggability and clinical significance. TCGA expression analysis versus corresponding normal tissue revealed that 19 of the 20 signature genes are overexpressed in HNSCC (Fig. S13A). Higher resolution analysis by utilizing a single-cell transcriptomic data set (scRNA-Seq) of 10 oral cavity carcinomas (GSE103322; Fig. S13B) uncovered that the majority of signature genes are either generally (e.g. Cyclophilin A (PPIA), β-tubulin (TUBB) and Peptidylprolyl Isomerase B (PPIB)), or more heterogeneously overexpressed (e.g. Tyrosine-protein kinase receptor UFO (AXL), Plasminogen Activator, Urokinase Receptor (PLAUR), Rac Family Small GTPase 2 (RAC2)) across the 1891 single HNSCC cells (Fig. S13B).

A more in-depth computational functional characterization through a correlation of expression profiles of our 20 signature genes with inferred signaling pathway activities using PROGENy (Pathway RespOnsive GENes for activity inference) revealed the strongest positive correlation for EGFR and MAPK activity in both bulk and scRNA-Seq from HNSCC patient samples (Fig. 7D). In general, OS signature genes exhibited increased negative correlations, for example, for the TRAIL-pathway. In contrast, PFS signature genes correlated more positively with a broad range of activated pathways including TNFα and NFкB signaling (Fig. 7D).

Finally, we integrated the deduced OS and PFS signature genes into the results of our kinase inhibitor screen conducting multiple network analyses. A STRING database analysis demonstrated a close linkage between the identified druggable kinases and 12 out of our 20 signature genes (Fig. 7E). Especially the PFS signature genes FOSL1, PLAU, PLAUR and ITGA5 clustered closely together and interconnected to EGFR-, cell cycle-, and EMT-related first neighbors. The highly effective SMI for PRKCA and PAK1-3 linked to each other and additional candidates like FYN via PLCγ (PLCG1). This integrated node is a direct substrate of the FGFR family. A concluding GeneMania interaction network emphasized the central role of EGFR based on predicted, physical and genetic interactions as well as co-expression, protein-domain and pathway commonalities (Fig. 7F). All identified druggable kinases whose inhibition was able to diminish or abrogate FGFRi-induced resistance share a direct interaction with EGFR together with six signature genes, including HBEGF and FOSL1. In summary, the in vitro derived transcriptomic FGFRi-induced resistance signature is transferrable to clinical cohorts. The two risk score signatures effectively prognosticate OS and PFS in HPV-negative HNSCC patients as well as PFS in the subcohort of radiotherapy-treated patients. Characterization of the signature genes revealed their broad overexpression in HNSCC and their correlation with high EGFR/MAPK and anti-apoptotic pathway activities. Interconnecting these signature genes to our previously identified kinase inhibitor targets unlocked potentially druggable connection nodes.

### EGFR acts as a key determinant in the adaptive resistance response to FGFRi

**Fig. 8:**
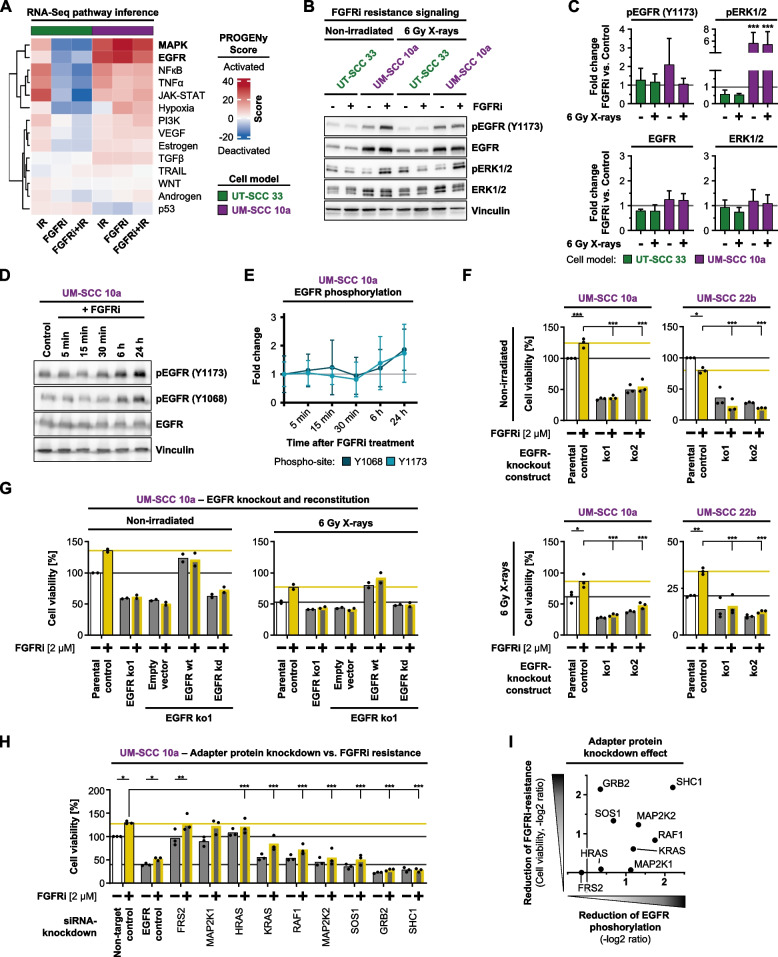
EGFR signaling essentially contributes to the protective FGFRi-induced resistance response. **A** Pathway activity inference derived from the three DEG comparison groups (IR, 6 Gy X-rays; FGFRi, FGFR inhibitor; FGFRi/IR, FGFR inhibitor plus 6 Gy X-rays) per cell model (*n* = 4) using PROGENy. Rows are clustered hierarchically. **B** Western blots of phosphorylated EGFR (Y1173) and ERK1/2 (Thr202/Tyr204) in whole cell lysates from 3D lrECM cell models treated as indicated. Vinculin served as loading control. Representative blots are shown. **C** Densitometric analysis of western blot results shown in ‘B’. Mean fold changes (± standard deviation) compared to corresponding non-irradiated/irradiated control are shown (*n* = 3). (Two-way ANOVA utilizing normalized densitometry data, Tukey multiple comparison test, ***p ≤ 0.001). **D** 24-h time kinetic of indicated EGFR phosphoforms in whole cell lysates from treated 3D lrECM UM-SCC 10a cell cultures. Vinculin served as loading control. Representative blots are shown. **E** Densitometric analysis of western blot data shown in ‘D’. Mean fold changes (± standard deviation) compared to the corresponding control are shown (*n* = 3). **F** Cell viability of CRISPR/Cas9-mediated EGFR-knockout cells (ko1, ko2) and corresponding controls after FGFRi treatment under non-irradiated and 6 Gy X-ray irradiated conditions. Bars represent mean cell viability (*n* = 3; two-way ANOVA; Tukey multiple comparison test; ***p ≤ 0.001, **p ≤ 0.01, *p ≤ 0.05). **G** Cell viabilities of UM-SCC 10a EGFR-knockout cells (ko1) reconstituted with either EGFR wild-type (wt) or kinase-dead (kd) constructs upon FGFRi treatment under non-irradiated and 6 Gy X-ray irradiated conditions. Parental cells and empty-vector-transduced cells were used as controls. Bars represent mean cell viability (*n* = 2). **H** Cell viability of UM-SCC 10a cells upon siRNA-mediated knockdown of indicated target genes alone or in combination with FGFRi treatment. Bars represent mean cell viability (*n* = 3; two-way ANOVA; Tukey multiple comparison test; ***p ≤ 0.001, **p ≤ 0.01, *p ≤ 0.05). Non-targeting siRNAs and DMSO were used as controls. **I** Effectiveness plot of adapter protein RNAi screen shown in ‘G’ (y-axis: cell viability; derived from Fig. 8G; x-axis: EGFR (Y1173) phosphorylation; derived from Fig. S15C, grey bars). Respective ratios to corresponding controls were calculated and -log2 transformed

Our presented observations determined EGFR as one of the central interconnectors between clinically relevant resistance signature genes and resistance-deactivating kinases, which are associated with the induction of partial EMT phenotype. To mechanistically investigate the role of EGFR, we first bioinformatically used the PROGENy database to obtain profiles showing strong activation of EGFR and MAPK signaling upon FGFR inhibition in UM-SCC 10a versus UT-SCC 33 cells (Fig. 8A, Fig. S14). In fact, this notion was confirmed by western blot analyses for EGFR and ERK (Fig. 8B-C) as well as in a 24-h phospho-EGFR kinetic under FGFRi exposure revealing a twofold elevation at 24 h (Fig. 8D-E). Accordingly, the generation of two EGFR knockout (ko) models derived from UM-SCC 10a and UM-SCC 22b cells provided further confirmation of both abrogation of FGFRi-induced cyto- and radioprotection as well as an expected decline in cell viability (Fig. 8F). Importantly, UM-SCC 10a EGFR-ko cells reconstituted with EGFR wild-type (wt) form showed FGFRi-related cytoprotection and radioprotection, whereas these effects were absent when reconstituted with an EGFR kinase dead (kd) form (Fig. 8G, Fig. S15A). Therefore, these findings evidently demonstrate EGFR as a key determinant of an adaptive cyto- and radioprotective response to inhibition of FGFR.

Apparently, FGFRs and EGFR interact mutually and/or cooperatively to induce EGFR phosphorylation when FGFR are inhibited. A knockdown screen of nine known essential adapter proteins on either receptor side allowed us to identify the interconnectors responsible for EGFR phosphorylation upon FGFR deactivation (Fig. 8H). Intriguingly, depletion of six out of nine adapter proteins accomplished a decrease in phospho-EGFR levels, with MAP2K2 (MEK2) and SHC1 being the top two candidates (Fig. S15B-C). These findings suggest specific signaling pathway activations of EGFR and its downstream signaling mediators after FGFR targeting.

## Discussion

Elucidating novel vulnerabilities for therapeutic interventions is paramount to curing cancer patients and preventing relapse. In this regard, key findings of our study on RTK/β1 integrin interactions and FGFR targeting in HNSCC encompass (a) FGFR inhibition elicits a cytotoxic/radiosensitizing-to-cytoprotective/radioprotective spectrum of responses, (b) additional β1 integrin inhibition only partially and cell model-dependently enhances the cytotoxic/radiochemosensitizing effects mediated by FGFR inhibition, (c) FGFR inhibition induces a marked adaptive resistance through an EGFR-driven pEMT in resistant cell models, (d) this pEMT resistance contains druggable kinases whose inhibition prevents the FGFRi-induced adaptive resistance response and enhances the FGFRi efficacy in certain cell models and organoids, and (e) contains specific signature genes with prognostic value for HNSCC patients. These results are highly relevant because (i) a transcriptomic response map for opposite directions of response to FGFR inhibition and irradiation has been constructed, (ii) a novel EGFR-driven pEMT resistance with (radio)protective properties has been described, and (iii) a broad network of potentially clinically relevant FGFRi and pEMT resistance-disabling targets was established for further exploration. Importantly, all experiments were performed under physiological 3D lrECM culture conditions, which were shown to resemble the growth and response behavior of HNSCC cells in vivo [13, 14].

Preclinical studies of concomitant EGFR/β1 integrin targeting in HNSCC documented the existence of certain cancer models refractory to this approach [4, 13]. This raised the question whether there exist other inhibitory RTK/β1 integrin approaches with higher coverage and thus effectiveness. Here, we focused on 10 RTK with available FDA-approved drugs and various oncogenic alterations in HNSCC. While β1 integrin targeting presented effective in reducing cell viability and enhancing radiosensitivity in a cell model-dependent manner, FGFR1-4 generally emerged as the most promising, β1 integrin-independent candidates regarding cytotoxicity and radiosensitization. In a more translational approach with the SMI Erdafitinib, the inhibitory antibody AIIB2 and cisplatin, responsive versus non-responsive groups were identified. This appears to be relevant as the phenotypically and genotypically heterogeneous 3D lrECM cell models investigated appear to cover the heterogeneity known from patient response profiles. Exploring in more detail at Erdafitinib dosing, we discovered a cyto- and radioprotective response pattern in certain HNSCC cell models. This appeared significant as the FGFR family has been shown to be a major driver of tumorigenesis in HNSCC. But only recently, FGFR-deactivating drugs, including Erdafinitib, have made significant clinical progress in other cancers and may thus represent new promising therapeutic opportunities in HNSCC [9, 15]. However, this receptor family is explored to a lesser extent in HNSCC, especially in combination with standard-of-care treatment approaches including irradiation and chemotherapy [8, 15].

Due to the fact that patients respond differently to SMIs, such as Erdafitinib, and therapy resistances will inevitably occur in the clinical setting, we focused on this adaptive response. Published reports on how to effectively overcome resistance to FGFR inhibition are scarce [16–19]. Studies in urothelial, hepatic cholangio and breast carcinomas examined various targeting strategies for mutated FGFR variants and adaptive feedback loops activated after FGFR deactivation. In HNSCC, Koole et al. proposed co-administration of Gefitinib in FGFRi-resistant HNSCC cells [20] in contrast to Singleton et al. who preferred ERBB2 instead of EGFR or cMET inhibition [21]. The present study addressed the underlying mechanisms of FGFRi-induced adaptive resistance and whether potent targets can be identified in the transcriptomic profiles mechanistically underlying this adaptive resistance. In addition to our previously discovered EGFR-driven EMT in HNSCC [22], the observations presented here add another facet to the repertoire of HNSCC cell responses to FGFR targeting and irradiation: an EGFR-driven pEMT phenotype is activated in FGFR-inhibited HNSCC cells. Due to its hybrid, metastable phenotype, pEMT has been shown to essentially enhance aggressiveness, CSC plasticity, resistance to radio(chemo)therapy and targeted therapy [10, 23–25]. Interestingly, and in contrast to our previous observations where EGFR either promoted proliferation or induced EMT [26], both features are found in the here described pEMT resistance response. The unprecedented extent of FGFRi-dependent cyto/radiation protection requires further investigation to avoid these effects in patients.

Indeed, our data demonstrate sensitivity to FGFR inhibition in some cell models. This is characterized by a reversal of the EMT phenotype to a MET phenotype and indicates a clear response to treatment. A similar FGFRi-induced MET was described by Nguyen et al. in HNSCC cell models [27]. The adaptive resistance with radioprotective properties is, on the other hand, a novel observation in HNSCC and strongly indicative of treatment failure, opening a window for therapeutic exploitation and highlighting the need for therapeutic intervention. EGFR and MAPK signaling pathway components as well as the EMT transcription factor SLUG (SNAI2; [25, 28, 29]) or the putative cancer stem cell marker CD44 [23, 30] may be considered, at least partially, as effective cancer targets or surrogate biomarkers. We corroborate the findings of others [17–19, 31–33] that EGFR is one of most effective targets in this pEMT resistance response. This appears as a key finding based on the fact that the majority of our identified druggable kinases and signature genes have direct interconnections with EGFR, supporting the reciprocal relationship between FGFR and EGFR. Additions to this panel are potential targets with available FDA-approved drugs like AXL, cMET, or diverse CDK [7, 34] as well as Fyn kinase and PAK1-3, both involved in EMT and connecting β1 integrins and RTK signaling in focal adhesion complexes [35–37]. PKC, one of the central signaling mediators downstream of numerous tyrosine kinase and G-protein coupled receptors like FGFR, integrins, EGFR, E-Cadherin amongst others, also emerged as potential candidate [38, 39]. These relationships have been confirmed by beneficial combination effects in HNSCC organoids, which are important translational models for the development of targeted therapies. Overall, it was of paramount importance to us that the observed transcriptomic resistance profiles induced by FGFRi primarily assisted us in the identification of therapeutic targets and secondarily appear to have a relationship with overall survival and progression-free survival of HNSCC patients.

Concerning prognostic signatures, work from others reported EpCAM and SLUG [40] as well as a 75-gene list as prognostic biomarker of HNSCC recurrence including molecular determinants of EMT and NF-κB activation for therapeutic intervention [41]. Van der Heijden et al. showed that RNA sequencing data from 174 HNSCC patients yielded a prognostic EMT signature that co-defines outcome for radiochemotherapy [42]. Detailed bioinformatic analyzes concluded that EMT markers together with certain focal adhesion proteins and integrins as well as long non-coding RNA regulatory mechanisms of specific cancer subtypes represent potential new biomarkers for HNSCC therapy [43, 44]. Our additions to these aspects, which were not directed at identifying a prognostic signature, were from potential druggable genes that are upregulated during the FGFRi-induced resistance response. Matching these genes with TCGA and validation cohorts revealed specific OS and PFS signatures with adverse effects on survival. In terms of standard treatment strategies for HNSCC, our PFS signature interestingly predicted PFS of irradiated patients. Functional validation in scRNA-HNSCC data sets and proteogenomic interaction databases highlighted the close relationship of the identified signature genes to important resistance kinases with EGFR as a central junction, whose deactivation was able to overcome resistance. The complementarity of functional drug screening and translation of bioinformatic signatures to clinical cohorts may delineate critical resistant signaling networks in HNSCC in the future. Preclinical and clinical data suggest that EGFR and FGFR influence each other and are part of effective adaptive feedback loops [45]. In addition to previous studies focusing on anti-EGFR therapy, consideration of dual FGFR and EGFR (or EGFR-related) targeting appears to be critical for optimizing therapy in HNSCC by effectively deactivating survival- and EMT-promoting bypass signals.

## Conclusion

In conclusion, our study shows that FGFR inhibition alone, in most cases without concomitant inhibition of β1 integrin, induces a broad cytotoxic/radiochemosensitizing-to-cytoprotective/radioprotective response in HPV-negative HNSCC cells. A transcriptomic response map indicates opposing EMT profiles in the most responsive/non-responsive 3D lrECM cell models connecting FGFRi-mediated radiosensitization to EMT reversal (MET). The protective effects consist of an extensive, previously undocumented EGFR-driven pEMT response, which shows therapeutic vulnerabilities for horizontal and vertical targeting approaches in cells and patient-derived organoids alike, and includes signatures with clinical relevance. Further mechanistic investigations and clinical validation of FGFR inhibition including the elucidation of adaptive resistance mechanisms and prognostic biomarker signatures are needed for the development of new personalized therapeutic approaches for HNSCC.

## Methods

### Cell models

The HNSCC cell models Cal33, FaDu, HSC4, SAS, UT-SCC 5, UT-SCC 8, UT-SCC 9, UT-SCC 14, UT-SCC 15, UT-SCC 24b, UT-SCC 33 and UT-SCC 50 were kindly provided by R. Grénman (Turku University Central Hospital, Finland). Additional HNSCC cell models were generously provided by H. Bier (UD-SCC 3, UD-SCC 8; University of Düsseldorf, Germany) and T. E. Carey (UM-SCC 10a, UM-SCC 11b, UM-SCC 14b, UM-SCC 17a, UM-SCC 17b, UM-SCC 22b; University of Michigan, USA). Cells were cultured in complete DMEM (cDMEM), consisting of Dulbecco’s modified Eagle’s medium (Sigma-Aldrich) supplemented with 10% fetal bovine serum (Sigma-Aldrich) and 1% non-essential amino acids (Sigma-Aldrich), at 37 °C in a humidified atmosphere containing 8.5% CO_2_. 3D culture conditions were accomplished by embedding cells into 0.5 mg/ml laminin-rich extracellular matrix (lrECM; Matrigel™, BD), as previously published [13]. The identity of all cell models has been authenticated by STR DNA profiling and tested negative for mycoplasma contamination. Additional information is listed in Table S1.

### Treatments

For β1 integrin blocking, the inhibitory monoclonal antibody AIIB2 (rat, IgG) was isolated from a human choriocarcinomal hybridoma as published [13]. Nonspecific rat IgG antibody (sc-2026, Santa Cruz Biotechnology) was used as control. Kinase inhibitors were dissolved in DMSO and applied at the indicated concentrations. Manufacturer´s information is listed in Table S1. Equal volumes of DMSO were used as control. The applied concentrations of AIIB2 (20 µg/ml), Erdafitinib (2 µM) and the chemotherapeutic cisplatin (CDDP, Hexal AG; 0.5 µM) were selected for low cytotoxicity (IC20, data not shown). Cells were irradiated at room temperature with 200-kV 6 Gy single X-ray doses filtered with 0.5 mm Cu using Yxlon Y.TU 320 (Yxlon Int. GmbH). The absorbed dose was monitored before exposure by a Duplex dosimeter (PTW Freiburg).

### RNAi-mediated knockdown screen

The siRNA transfection was performed as previously described [46] by using the indicated combinations of ON-TARGETplus SMARTpool™ siRNAs against the respective targets or non-targeting control siRNA (both Horizon Discovery). After 24 h, cells were plated for 3D assays or harvesting of whole cell lysates for western blot analyses was performed.

### EGFR-knockout cell model generation

To knockout EGFR in two human HNSCC lines (UM-SCC 10a and UM-SCC 22b), two gRNAs using the Synthego CRISPR design tool (https://www.synthego.com/products/bioinformatics/crispr-design-tool) were designed. The two gRNAs – g1: 5’-TGAGCTTGTTACTCGTGCCT-3’, g2: 5’-GAGTAACAAGCTCACGCAGT-3’– were cloned into pL.CRISPR-puro vectors as previously described [47]. To produce the virus, 20 million HEK293T cells were seeded in T175 cell culture flasks in DMEM (Gibco) supplemented with 10% FBS (Gibco) and 1% penicillin/streptomycin (DMEM + +). Cells were transfected the day after by preparing and mixing two reactions for 15 min at RT: the first consisted of 36 μg lentiviral plasmid (pL.CRISPR-puro), 21.6 μg psPAX2 plasmid, and 7.2 μg pMD2.G plasmid in 2 ml of DMEM and the second consisted of 120 μL polyethyleneimine (PEI) in 2 ml of DMEM. After the reactions were mixed, 17 ml DMEM +  + was added, and the total mix (21 ml) was added carefully on top of cells. The media was exchanged on the next day (15 ml) and the supernatant was collected 48 h thereafter. After collection, the supernatant was passed through 0.45 μm filter units (Millex) and concentrated on filter tubes (Amicon Ultra-15, Merck) down to the volume of 200 μL (centrifugation 1500 g, 45 min). The virus was mixed with cDMEM (see above) and cells were spinoculated in 24-well plates at 700 g for 1 h. Forty-eighth hours after spinoculation, cells were split and selected in 2 μg/ml puromycin for five days. Genomic DNA was isolated and the PCR was performed around the site of each gRNA cut using primers F: 5’- ATGGGTGAGTCTCTGTGTGG-3’ and R: 5’- TGGTCAGGGATAAACGTCAGT-3’. Resulting bulk PCR Sanger sequencing files were analyzed using the Synthego ICE analysis tool (https://www.synthego.com/products/bioinformatics/crispr-analysis) and the most efficient gRNA, gRNA g1, was chosen for the downstream experiments (-ko1 cell models). psPAX2 plasmid (Addgene #12260) and pMD2.G plasmid (Addgene #12259) were kindly provided by D. Trono (Swiss Federal Institute of Technology Lausanne, Switzerland).

### EGFR constructs and transductional reconstitution

The plasmid pECFP-N1-hEGFR was kindly provided by L. E. Samelson, (NIH, Bethesda, USA). We designed a forward primer with a KpnI restriction site and a reverse primer with NheI restriction site to clone the hEGFR cDNA into the pL.OE vector [47], where cDNA is co-expressed with EGFP. The gRNA g1 protospacer adjacent motif (PAM) in EGFR cDNA was mutated, resulting in our final pL.OE-EGFR(C2654T) vector. This mutation and the subsequent EGFR kinase domain deletion (kd) were performed using QuickChangeII XL Site directed Mutagenesis (Agilent). The mutated sites were confirmed by sequencing. All utilized cloning and mutagenesis primers are listed in Table S1. Virus production and transduction of UM-SCC 10a EGFR-ko1 cells was performed as described above with an additional ultracentrifugation step and as published [47]. After 5 days, transduced cells were plated for 3D cell viability assays.

### 3D cell viability assay

5 × 10^3^ cells were embedded in 0.5 mg/ml lrECM in 96-well plates as reported [13]. If applicable, inhibitors or antibodies and their respective controls (DMSO, IgG) were applied after 22 h, followed by CDDP one hour later. Irradiation was performed after one additional hour of incubation and cells subsequently grew for 96 h. Cell viability was measured using the 3D CellTiter-Glo® 3D Assay (Promega) according to the manufacturer’s instructions. The results are partly presented as enhancement ratios (ER), which describes the average viability of the respective control (non-irradiated/irradiated) divided by the average viability after the indicated treatment.

### 3D colony formation assay

Cells were embedded into 0.5 mg/ml lrECM in 96-well plates as described [13]. Treatments were identical to the ones described under ‘3D cell viability assays’. After a cell model-dependent growth period, cell colonies were fixed with 9% formaldehyde solution in PBS. Microscopic, focus-stacked image acquisition was performed using Cytation 5 reader (BioTek), followed by colony counting and area analysis in Fiji [48]. All colonies with a minimum diameter of 50 µm were included in the analysis.

### Western blot analysis

Western blot analysis on whole cell lysates from 3D lrECM cultured cells was performed as published [13, 49]. For basal protein level assessment, 1 × 10^6^ cells were grown in 0.5 mg/ml lrECM for 24 h prior to lysis. Whole cell 3D lysates from treated cells were harvested 4 days after treatment, if not indicated differently. The utilized antibodies are described in Table S1. Densitometry analysis was performed in Fiji or Fusion Software (Vilber Lourmat GmbH). All samples were normalized to their corresponding loading control and phosphoforms were further normalized to total protein expression.

### Thawing of cryopreserved HNSCC organoids

Patient-derived HNSCC organoids utilized in this study were previously established and cryopreserved [50]. Supplementary culture information is presented in Table S1. Cryovials containing the organoids were retrieved from liquid nitrogen and thawed in a water bath maintained at 37 °C. Subsequently, the organoid suspension was added into 10 ml of advanced DMEM-F12 medium (Life Technologies) supplemented with 1 × GlutaMAX (adDMEM/F12; Life Technologies), Penicillin–streptomycin (Life Technologies), and 10 mM HEPES (Life Technologies) (designated as + / + / + medium). The thawed organoids were centrifuged at 400 g for 5 min at 4 °C, and the supernatant was aspirated. The resulting organoid pellet was suspended in 20 µl of ice-cold 70% solution of 10 mg/ml Cultrex growth factor-reduced BME type 2 (Trevigen) in + / + / + medium. The density of organoids was assessed under a microscope, and additional 70% BME suspension was added if necessary to achieve the desired concentration. The organoid suspension was then plated in small droplets (10–15 µl) onto pre-warmed 24- or 12-well suspension plates. The plates were inverted and incubated at 37 °C for 15–20 min to allow the organoid-BME suspension to solidify. Following solidification, pre-warmed culture medium was gently added to the wells, and the plates were returned to a 37 °C, 5% CO_2_ incubator. The medium was changed every two to three days, and depending on growth rate, organoids were passaged every 7–10 days. In this study, two types of previously described culture media were used for HNSCC organoids depending on the line (Table S1): Head and Neck (HN) medium [51] or Cervical Squamous Cell Carcinoma (SCC) medium [52].

### Passaging of HNSCC organoids

Organoids embedded in BME droplets were disrupted by suspending the content of the wells using a P1000 pipette and transferring it to a 15 ml falcon tube. The volume was adjusted to 15 ml using + / + / + medium and then centrifuged at 400 g for 5 min. Pellets obtained were resuspended in 2 ml TrypLE Express (Life Technologies) and incubated at 37 °C for 5–15 min. The digestion process was monitored under a microscope, and mechanical shearing using a P1000 pipette was performed intermittently until the organoids were disrupted into single cells. The digestion was stopped by topping up the tubes with + / + / + medium, followed by centrifugation (400g, 5 min). After removing the supernatant, the cells were resuspended in 70% BME in + / + / + medium. Organoid density was rechecked under the microscope before plating; if too dense, additional 70% BME in + / + / + was added. Domes of 10–20 µl were plated on pre-heated suspension culture plates (Greiner), inverted, and incubated at 37 °C for 15–20 min for BME solidification. Once solidified, pre-warmed culture medium supplemented with 10 µM Y-27632 was added, and the plates were incubated in a 37 °C, 5% CO_2_ incubator.

### Drug screening on HNSCC organoids

The biobanked organoids were thawed and expanded using the protocols mentioned above. Two days before dispensing for drug screening, organoids were passaged and cultured in HN or Cervical SCC medium depending on the line. On the day of dispensing, 1 mg/ml Dispase II (Sigma-Aldrich) was added to each well with organoids, and incubated for 30 min at 37 °C. Subsequently, BME domes were disrupted, and the organoids were collected and transferred to 15 ml falcon tubes. Dispase II was removed by topping up with + / + / + medium and centrifuged at 400 g for 5 min at 4 °C. Following supernatant removal, the pellets were suspended in 10 ml + / + / + medium and centrifuged again. The resulting organoid suspensions were filtered through 70-μm nylon cell strainers (Greiner Bio-One, EASYstrainer™ small), and the number of organoids in the flow-through was counted using a KOVA™ counting chamber (Fisher-Scientific). Organoids were resuspended at a density of 25,000 organoids/ml in 5% BME/ice-cold HN medium. The organoids were dispensed into a 384-well plate (Corning) using a Multi-drop Combi Reagent Dispenser (Thermo Scientific), plated in triplicate for inhibitor characterization and sextuplicates for drug combinations experiments. Drugs were added using a Tecan D300e Digital Dispenser, with all wells normalized for the amount of drug solvent (DMSO) used. The plates were sealed (BreathEasy stickers, Merck) and placed in a 37 °C/5% CO_2_ incubator until the drug screen readout. Plates designated for radiotherapy received irradiation (6 Gy X-rays) approximately 24 h after drug dispensing using a linear accelerator (Elekta Precise Linear Accelerator 11F49, Elekta). Plates were submerged in water at room temperature. After incubation with drugs for 5 days, cell viability was measured using the 3D CellTiter-Glo® 3D Assay (Promega) according to the manufacturer’s instructions. Based on our inhibitor characterization, we utilized for subsequent drug combination experiments the approximate IC20 concentrations of FGFRi (Erdafitinib; 1,5 µM), EGFRi_1 (Lapatinib; 2 µM), PKCi (Ro 31–8220 Mesylate, 0.33 µM) and PAK1-3i (FRAX597; 0.1 µM) (the solvent DMSO served as controls).

### Whole exome sequencing

DNA isolation of 3D lrECM cultured UT-SCC 5 cells was similarly performed as previously published [53]. Raw whole exome sequencing (WES) data of SAS, FaDu and UT-SCC 15 cells was previously sequenced (75 bp paired-end) [53], UT-SCC 5 cells were newly sequenced utilizing the same methodology (100 bp paired-end). Both were quality checked using FastQC (v0.11.4) and adapter removal and trimming of reads was done by TrimGalore (v0.4.2; Both: https://www.bioinformatics.babraham.ac.uk/). Mapping of reads against the human genome reference sequence (GRCh37 release 13) was performed by BWA-MEM (v0.7.13) [54] with standard settings and duplicates were marked using Samblaster (v0.1.24) [55]. Mapped reads were locally aligned with the Genome Analysis Toolkit (GATK 3.5, tools: RealignerTargetCreator, IndelRealigner, BaseRecalibrator, PrintReads) [56]. Alignment summary metrics were determined with Picard tools (v1.141, http://broadinstitute.github.io/picard/) and SAMtools (v1.3) [57]. Mutect2 was used for variant calling [58]. Additional filtering and annotation of the predicted variants was done with Annovar (v1Feb2016) [59]. The predicted variants were annotated with ClinVar, COSMIC (v.94), avsnp150 and gnomAD_exome variant information. For each cell model, only exonic, protein-altering mutations that were independently predicted in all three technical replicates were considered and filtered on the basis of COSMIC listing or low allele frequencies (gnomAD_exome_ALL < 1%) for subsequent evaluation (Table S2).

### Whole exome copy number prediction

Mapped reads against the human genome reference sequence (GRCh37 release 13) from the whole exome sequencing analysis of the individual HNSCC cell models were used to determine putative deletions or duplications affecting exomes of selected genes. For each HNSCC cell model, reads mapped to the exomes of selected genes (ALK, AXL, DDR1, FGFR1, FGFR2, FGFR3, FGFR4, ITGB1, MET, RET, ROS1) were counted (R function countBamInGRanges from package exomeCopy). Because a normal reference of exome counts was not available, average counts per exome analyzed were calculated across cell models. This was done separately for the previously sequenced HNSCC cell models (FaDu, SAS, UT-SCC 15) and the newly sequenced HNSCC cell model (UT-SCC 5) to account for batch effects of both individual sequencing runs. Next, log2-ratios for the exome read counts were computed for each cell model in relation to the corresponding average exome count reference. This made it possible to determine, for each cell model, exomes that differ greatly from the average cell model, indicating possible deletions (log2 ratios significantly below zero) or possible duplications (log2 ratios significantly greater than zero). The log2-ratio exome copy number profiles of the genes were further visualized by heatmap representations (R function heatmap.3 with cluster method ward.D2 and euclidean distance as measure between two cell models). The obtained exome copy number profiles were cell model-specific and highly reproducible for the three independent replicates that were available for each cell model.

### RNA extraction

Samples for RNA-sequencing (RNA-seq) were cultured and treated with the FGFR inhibitor Erdafitinib and irradiation as described under ‘3D cell viability assay’. Multiple technical replicates of 1 × 10^5^ cells were seeded per cell model with or without treatment. Five days after seeding, technical replicates were harvested and pooled for each treatment group [60]. RNA was extracted using the NucleoSpin RNA kit (Machery-Nagel), followed by RNA-integrity measurement (4200 TapeStation, Agilent).

### RNA sequencing

Only total RNA with RNA-integrity numbers ≥ 9.5 was used. mRNA was isolated from 370 ng DNAse treated total RNA using the Next rRNA depletion Kit (New England Biolabs) according to the manufacturer’s instructions. Samples were subjected to the workflow for strand specific RNA-Seq library preparation (Next Ultra II Directional RNA Library Prep, New England Biolabs). For ligation, custom adaptors were used (Adaptor-Oligo 1: 5'-ACA CTC TTT CCC TAC ACG ACG CTC TTC CGA TCT-3', Adaptor-Oligo 2: 5'-P-GAT CGG AAG AGC ACA CGT CTG AAC TCC AGT CAC-3'). After ligation adapters were depleted by an XP bead purification (Beckman Coulter) by adding bead in a ratio of 1:0.9. Unique dual indexing was done during the following PCR enrichment (12 cycles) using custom amplification primers carrying the index sequence indicated with ‘NNNNNNN’ (Primer 1: AAT GAT ACG GCG ACC ACC GAG ATC TAC ACT CTT TCC CTA CAC GAC GCT CTT CCG ATC T, Primer 2: CAA GCA GAA GAC GGC ATA CGA GAT NNNNNNNN GTG ACT GGA GTT CAG ACG TGT GCT CTT CCG ATC T). After two more XP beads purifications (1:0.9), libraries were quantified using the Fragment Analyzer (Agilent). Libraries were equimolarly pooled before sequencing them on an Illumina NovaSeq 6000 system in 100 bp paired-end mode to a depth of at least 40 million fragments.

### Differential expression analysis

FastQC (http://www.bioinformatics.babraham.ac.uk/) was used to perform a basic quality control of the resulting sequencing data. RNA-SeQC 2 checked quality after alignment [61]. Fragments were aligned to the human reference genome hg38 with support of the Ensembl 104 splice sites using the aligner gsnap (v2020-12–16) [62]. Fragments per gene and samples were obtained based on the overlap of the uniquely mapped fragments with the same Ensembl gene annotation using featureCounts (v2.0.1) [63]. Normalization of raw fragments based on library size and testing for differential expression between the different cell types/treatments was done in R (DESeq2, v1.36.0). The interrelation between biological replicates and conditions was explored by hierarchical clustering and principal component analysis (PCA) of the top 5000 genes showing highest variance (R, PCAtools, v2.12.0). To identify differential expressed genes (DEG), counts were fitted to the negative binomial distribution and genes were tested between conditions using the Wald test of DESeq2. Resulting *p*-values were corrected for multiple testing with the using Independent Hypothesis Weighting (v1.24.0) [64]. Genes with a maximum of 5% false discovery rate (padj ≤ 0.05) were considered as significantly differentially expressed.

### Functional characterization, gene sets and pathway inference

The DEG between treatment groups were implemented in overrepresentation analyses for Gene Ontology (GO), Kyoto Encyclopedia of Genes and Genomes (KEGG) and Reactome databases (R, clusterProfiler, v4.8.1). Gene set enrichment analyses (GSEA) were performed on ranked DEG data sets (R, fGSEA, v1.26.0) using hallmark gene sets of the Molecular Signature Database (MSigDB) and others [22, 25, 65–69] listed in Table S3. Potentially druggable genes were retrieved from the Drug-Gene Interaction Database (DGIdb 4.0; https://dgidb.org/) and overlapped with the human kinome (http://kinhub.org/) to obtain the gene set of druggable kinases. PROGENy (Pathway RespOnsive GENes for activity inference) [70] was utilized to compute the activity across 14 major cellular pathways in cell model and clinical (sc)RNA-Seq data sets in R (decoupleR, 2.6.0). The top 500 PROGENy model gene weights were used, and regulatory activities were calculated by normalized weighted mean. For network analyses, STRING (https://string-db.org/) and GeneMANIA (http://genemania.org) databases were utilized in Cytoscape (v3.9.1). Box- and volcano plots (ggplot2, v3.4.2), Heatmaps (ComplexHeatmap, v2.16.0), and Multi-comparison GSEA plots (Biokit, v0.1.1) were visualized in R.

### Mutational, RNA expression and patient survival datasets

Clinical RNA expression data from the 2018 TCGA-HNSCC cohort (log2 normalized, generated by the TCGA Research Network: https://www.cancer.gov/tcga) was acquired from *Xena* (https://xenabrowser.net/). Corresponding clinical information and curated somatic mutation data were extracted from cBioPortal (https://cbioportal.org/). We selected for HPV-negative patients, resulting in *n* = 415 primary tumor samples. Additionally, microarray-based HPV-negative HNSCC cohorts were obtained from Fred Hutchinson Cancer Research Center (FHCRC, *n* = 97) and MD Anderson Cancer Center (MDACC, *n* = 74) via Gene Expression Omnibus (GEO) with accession numbers GSE41613 and GSE42743, respectively. Only patients with available clinical endpoints and target gene expression were implemented in further analyses. Pre-processed scRNA-sequencing data was retrieved from GEO (GSE103322) and filtered for the ten patients samples with the highest fraction of malignant cells [25] in R (Seurat, v4.3.0).

### Patient survival analysis

A druggable adaptive resistance gene set was derived from the response profile of UM-SCC 10a cells towards FGFR inhibition, listed in Table S3. The focus was put on upregulated DEG (log_2_FC ≤ 0.5) above minimum expression (normalized counts > 10), which were not similarly upregulated in sensitive UT-SCC 33 cells, intersected with potentially druggable genes (DGIdb 4.0) and increased upon FGFR inhibitor application on top of irradiation. Overall survival (OS) and progression-free survival (PFS) were the main clinical endpoints in this study and TCGA-HNSCC the main test cohort. Univariate Cox regression model was employed using sklearn (v1.2.2) and sksurv (v0.21.0) packages in Python (v3.11.4) to analyze the adaptive resistance gene set. Genes were filtered based on their upregulated (HR > 1) or downregulated (HR < 1) status, with a significance threshold of *p*-value ≤ 0.05. Subsequently, a multivariate Cox regression model was applied, and risk scores were calculated by summing up coefficient-weighted gene expression values for each individual patient. The median risk score from the TCGA HNSCC cohort was utilized for prognostic stratification. Survival analysis and visualization were performed using the CoxPHSurvivalAnalysis (sksurv, v0.21.0), CoxPHFitter, and KaplaMeierFitter functions (lifelines, v0.27.7). To validate the findings, the resulting signatures from OS and PFS regression analyses were applied to FHCRC and MDACC HNSCC patient cohorts. These cohorts only contain survival data for the clinical endpoint OS. Patient stratification into high- and low-risk groups are listed for all three HNSCC cohorts in Table S3. Prior to training and validating prognostic Cox regression models, the RNA expression values were log2 transformed and scaled. The predictions for 5-year OS and PFS of all models were visually represented by Kaplan–Meier curves.

### Experimental data analysis and statistics

Experimental data from cell models are presented as the mean of three independent biological experiments (indicated as *n*) ± range or standard deviation. Statistical analyses of cell viability data were performed in Prism 8 (GraphPad Prism Software Inc.) by implementing raw luminescence data in a two-way ANOVA (randomized block ANOVA) followed by Dunnett’s post-hoc test, if treatments were solely compared to control, or Tukey post-hoc test if treatments were additionally compared among each other. Similar statistical analyses were applied to loading-control normalized western blot densitometry. Western blot data from phosphorylated forms of proteins was additionally normalized to the corresponding total protein signal. HNSCC organoid inhibitor characterizations were performed in Prism 8 by non-linear regression analysis [51]. Colony formation assay results were normalized to the seeded cell number and statistically evaluated by a two-tailed paired Student’s t-test. Combinatory effects were assessed by the highest-single-agent (HSA) approach [71]. Potential synergism was calculated by the Bliss definition of drugs independence [72]. TCGA expression differences between tumor and normal samples were compared by two-tailed unpaired t-test. The statistical analysis of correlating PROGENy pathway activity to clinical RNA-Seq datasets was performed in R via corr.test function with Holm *p*-value adjustment (psych, v2.3.6). Patient survival analyses were performed in Python (v3.11.4). Other bioinformatical analyses were performed in R (v4.3.0). *P*-values of less than 0.05 were considered statistically significant and depicted as: ***p ≤ 0.001, **p ≤ 0.01, *p ≤ 0.05.

### Supplementary Information

Below is the link to the electronic supplementary material.

**Supplementary material 1.**

**Supplementary material 2.**

**Supplementary material 3.**

**Supplementary material 4.**

## Data Availability

The datasets generated and analyzed during the current study are available from the corresponding author on reasonable request. All analyzed clinical datasets analyzed are publicly available and were disclosed in Methods.

## Abbreviations

AIIB2: Anti-β1 integrin antibody AIIB2;
CDDP: Cisplatin
EMT: Epithelial-to-Mesenchymal Transition
ER: Enhancement ratio(s)
FC: Fold change
FGFRi: FGFR inhibitor Erdafitinib
FHCRC: Fred Hutchinson Cancer Research Center
GEO: Gene Expression Omnibus
GO: Gene Ontology database
GSEA: Gene Set Enrichment Analysis
HNSCC: Head and neck squamous cell carcinoma
HPV: Human papillomaviruses
IC: Inhibitory concentration
IR: 6 Gy X-Ray irradiated
KEGG: Kyoto Encyclopedia of Genes and Genomes
kd: Kinase dead construct
ko: Knockout construct
lrECM: Laminin-rich Extracellular Matrix
MDACC: MD Anderson Cancer Center
MET: Mesenchymal-to-Epithelial Transition
NES: Normalized enrichment score
OS: Overall survival
p.adj/adj. *p*-value: Adjusted *p*-value
PCA: Principal Component Analysis
PFS: Progression Free Survival
RNA-seq: RNA-sequencing
RTK: Receptor Tyrosine Kinase
scRNA-seq: Single cell RNA-sequencing
sig.: Significance
SMI: Small molecule inhibitor
Wt: Wild-type
Y: Tyrosine
