# Supplementary material for: Resistance of HNSCC cell models to pan-FGFR inhibition depends on the EMT phenotype associating with clinical outcome
Source: Mol Cancer. 2024 Feb 21;23:39. doi: 10.1186/s12943-024-01954-8 (PMC10880239; doi:10.1186/s12943-024-01954-8)
Supplement: Supplementary file 1 — Supplementary material 1. [file 12943_2024_1954_MOESM1_ESM.pdf]

Supplementary Information

Resistance of HNSCC cell models to pan-FGFR inhibition depends on the EMT phenotype associating with clinical outcome

Felix Broghammer [1], Irina Korovina [1,2], Mahesh Gouda [3], Martina Celotti [4], Johan van Es [4], Inga Lange [1], Cornelia Brunner [5], Jovan Mircetic [6,7], Robert P. Coppes [8,9], Olivier Gires [2], Andreas Dahl [10], Michael Seifert [11,12], Nils Cordes [1,2,6,12,13] *

1 OncoRay - National Center for Radiation Research in Oncology, Faculty of Medicine Carl Gustav Carus, Technische Universität Dresden, 01307 Dresden, Germany;

2 Helmholtz-Zentrum Dresden-Rossendorf (HZDR), Institute of Radiooncology - OncoRay, 01328 Dresden, Germany;

3 Department of Otorhinolaryngology, Head and Neck Surgery, Ludwigs-Maximilians-University University Hospital, 81377 Munich, Germany;

4 Hubrecht Institute, Royal Netherlands Academy of Arts and Sciences (KNAW) and University Medical Center Utrecht, 3584 CT, Utrecht, the Netherlands;

5 Department of Otorhinolaryngology, Ulm University Medical Center, 89075 Ulm, Germany;

6 German Cancer Consortium, Partner Site Dresden: German Cancer Research Center (DKFZ), 69120 Heidelberg, Germany;

7 Mildred Scheel Early Career Center (MSNZ) P2, Medical Faculty and University Hospital Carl Gustav Carus, Technische Universität Dresden, 01307 Dresden, Germany;

8 Department of Biomedical Sciences of Cells and Systems, Section of Molecular Cell Biology, University Medical Center Groningen, University of Groningen, 9713 Groningen, The Netherlands;

9 Department of Radiation Oncology, University Medical Center Groningen, University of Groningen, 9713 Groningen, The Netherlands;

10 DRESDEN-Concept Genome Center, Center for Molecular and Cellular Bioengineering, Technische Universität Dresden, 01307 Dresden, Germany

11 Institute for Medical Informatics and Biometry (IMB), Faculty of Medicine Carl Gustav Carus, Technische Universität Dresden, 01307 Dresden, Germany;

12 National Center for Tumor Diseases (NCT), Partner Site Dresden, German Cancer Research Center (DKFZ), 69192 Heidelberg, Germany;

13 Department of Radiotherapy and Radiation Oncology, University Hospital Carl Gustav Carus, 01307 Dresden, Germany

***Corresponding Author:** Prof. Dr. Nils Cordes, Nils.Cordes@OncoRay.de

Additional file 1: Supplementary figures

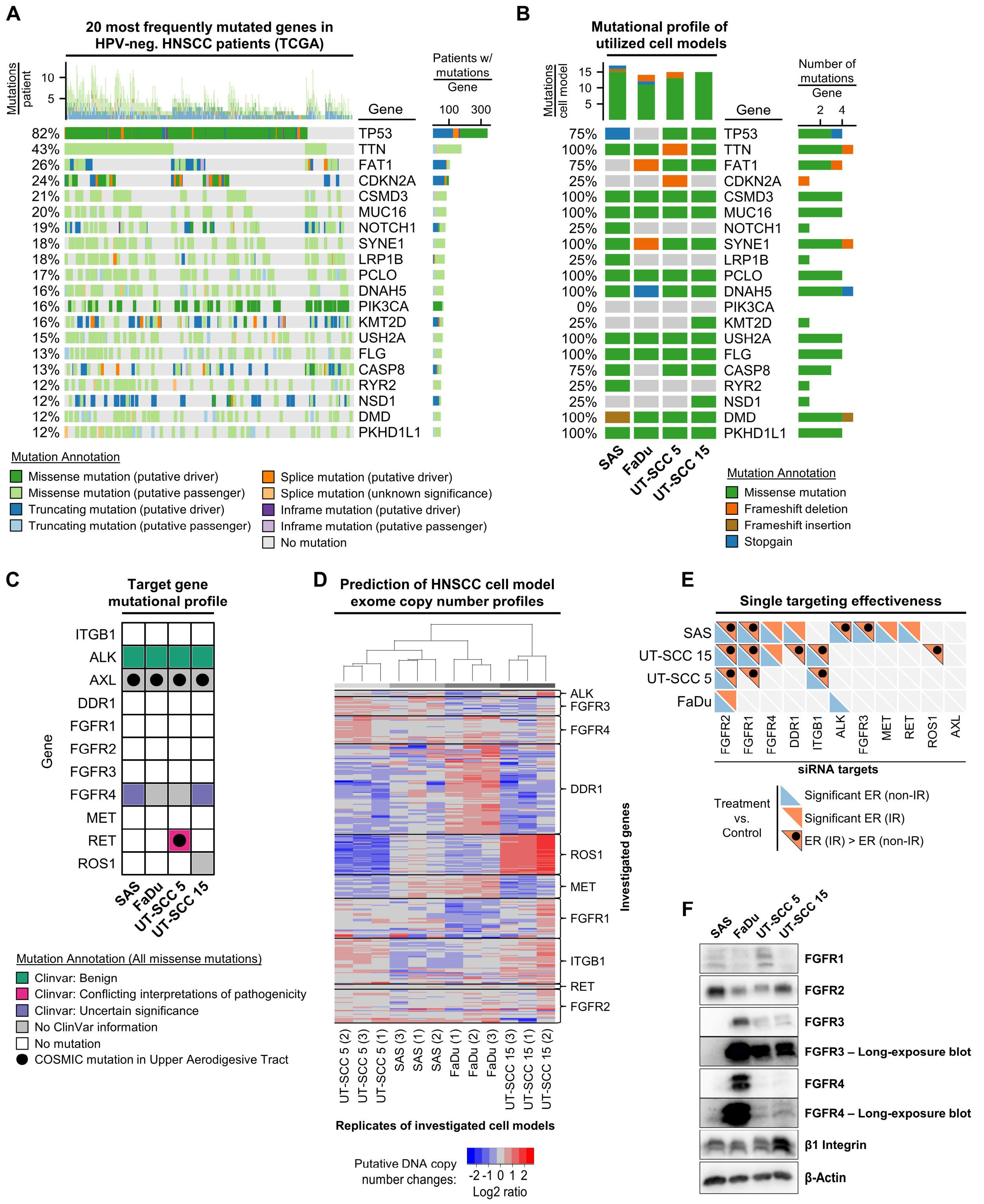

Fig. S1 Mutational data and protein expression of selected targets from the RNAi screen in the indicated cell models. **A** OncoPrint diagram of the 20 most frequently mutated genes of the HPV-negative HNSCC TCGA cohort (*n* = 415). Putative driver mutations were annotated by cBioPortal. Percentage values indicate the frequency of the alterations in all patients and were used for row ordering; column order represents mutual exclusivity across genes. **B** OncoPrint diagram of the mutational profile of the tested cell models. The 20 most frequently mutated genes of HPV-negative HNSCC patients (TCGA) were selected. Complete exome mutational information for the selected genes is presented in **Table S2**. **C** Mutational data of selected RTK target genes in the indicated cell models. All are classified as missense mutations; annotation includes ClinVar information and COSMIC occurrence in “Upper Aerodigestive tract” (v94). Complete exome mutational information for the selected target genes is presented in **Table S2**. **D** Log2-ratio heatmap of specific exome copy number profiles from SAS, FaDu, UT-SCC 5 and UT-SCC 15 cell models. Log2-ratios quantify putative DNA copy number changes in a specific cell model relative to the average counts across all cell models. Low log2-ratios (clearly < 0) indicate potential deletions affecting specific exomes, values around zero (grey) indicate exomes with unchanged copy numbers, and high log2-ratios (clearly < 0) indicate potential duplications affection specific exomes of a gene. **E** Summarizing diagram indicating significant cytotoxic effects of single RTK knockdowns at non-irradiated and 6 Gy X-ray irradiated conditions (IR) across the four cell models (derived from **Fig. 1D-E** and **Fig. S2**). A greater mean ER under IR (ER (IR) > ER (non-IR)), indicated by black dots, represents radiosensitization. **F** Western blot analysis of basal FGFR and β1 integrin expression from whole cell lysates of indicated 3D lrECM cell models. β-actin served as loading control. Representative blots from three independent experiments are shown, supplemented with corresponding long-exposure blots for low intensity markers.

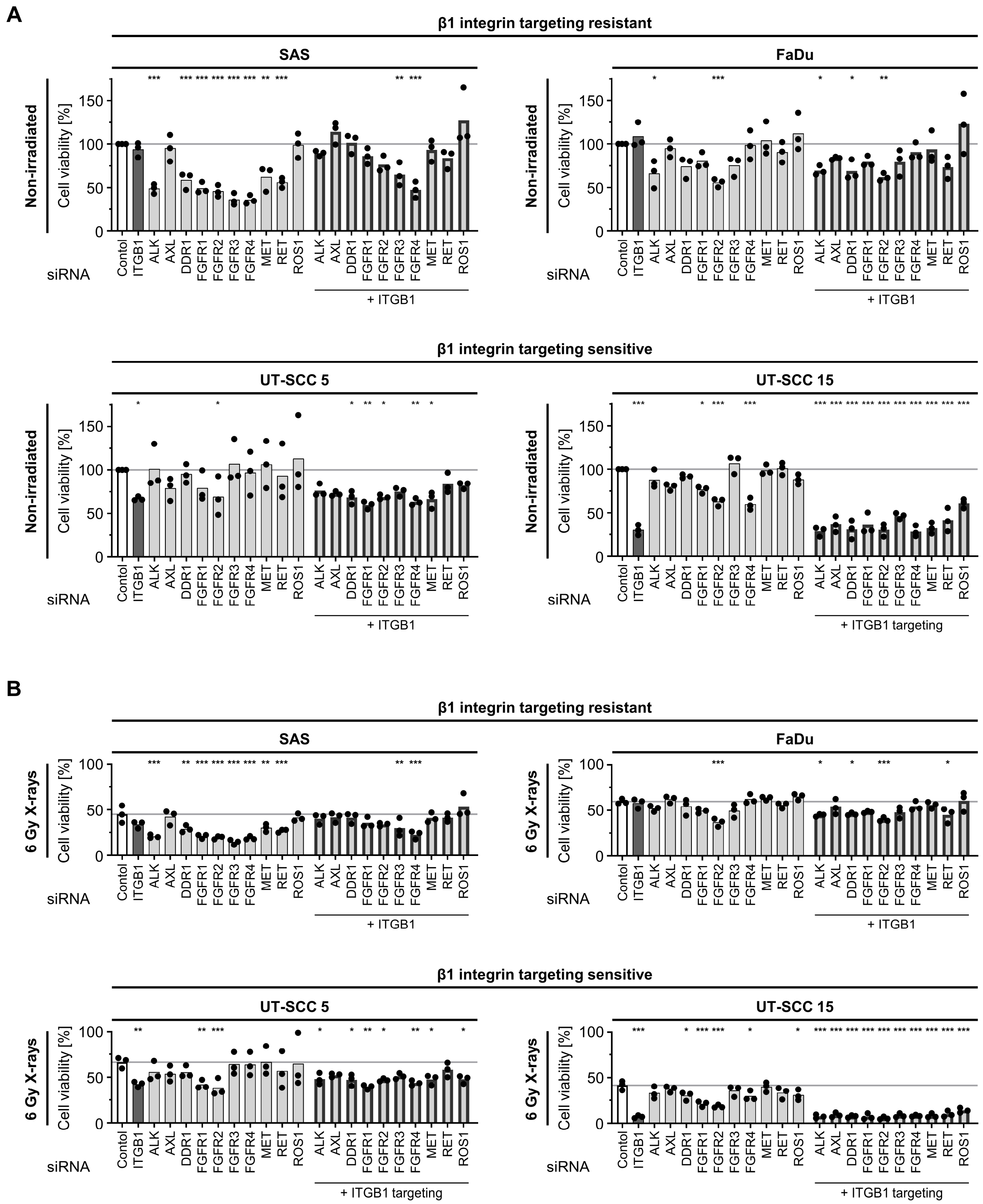

Fig. S2 Cell viability data of our RNAi screen. **A** Cell viability of indicated 3D lrECM grown HNSCC models upon single or double siRNA-mediated knockdowns of 10 RTK and β1 integrin (n = 3; mean; two-way ANOVA; Dunnett's multiple comparison test to corresponding controls; ***p ≤ 0.001; **p ≤ 0.01; *p ≤ 0.05). Non-targeting siRNAs were used as controls. **B** Cell viability of 6 Gy

X-rays irradiated 3D lrECM HNSCC models upon single or double siRNA-mediated knockdowns of 10 RTK and β1 integrin. Normalization was performed to non-irradiated control (*n* = 3; mean; two-way ANOVA; Dunnett's multiple comparison test to corresponding irradiated controls; ***p ≤ 0.001; **p ≤ 0.01; *p ≤ 0.05). Non-targeting siRNAs were used as controls.

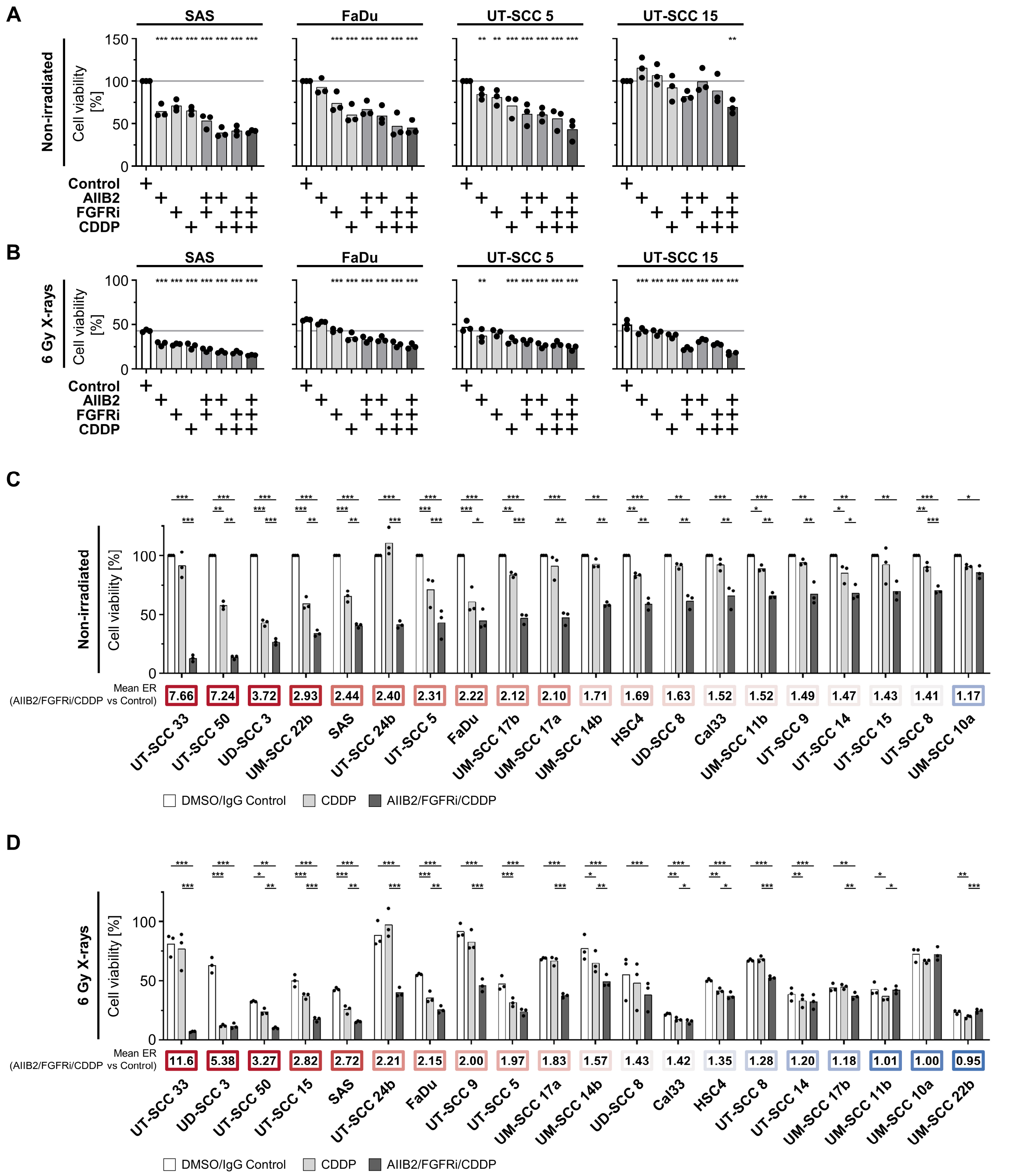

Fig. S3 Radiochemosensitizing potential of FGFR and β1 integrin targeting in various HNSCC cell models. **A** Cell viability of 3D lrECM HNSCC models upon indicated treatment combinations. DMSO/IgG were used as control (*n* = 3; mean; two-way ANOVA; Dunnett's multiple comparison

test to corresponding controls; ***p ≤ 0.001; **p ≤ 0.01; *p ≤ 0.05). **B** Cell viability of 3D lrECM HNSCC models upon indicated treatment combinations plus a single dose of 6 Gy X-rays. DMSO/IgG were used as control (*n* = 3; mean; two-way ANOVA; Dunnett's multiple comparison test to corresponding irradiated controls; ***p ≤ 0.001; **p ≤ 0.01; *p ≤ 0.05). **C** Cell viability of 20 HPV-negative 3D lrECM HNSCC cell models normalized to corresponding DMSO/IgG control upon single CDDP or triple AIIB2/FGFRi/CDDP treatment (*n* = 3; two-way ANOVA; Tukey multiple comparison test; ***p ≤ 0.001, **p ≤ 0.01, *p ≤ 0.05). Enhancement ratios (ER) of AIIB2/FGFRi/CDDP vs corresponding controls are shown and determine the cell models x-axis order. **D** Cell viability of 20 HPV-negative 3D lrECM HNSCC cell models normalized to corresponding DMSO/IgG control upon single CDDP or AIIB2/FGFRi/CDDP triple treatment plus 6 Gy X-ray irradiation (Two-way ANOVA, Tukey multiple comparison test, ***p ≤ 0.001; **p ≤ 0.01; *p ≤ 0.05). ER of AIIB2/FGFRi/CDDP/irradiation vs corresponding irradiated controls are shown and determine the cell models x-axis order. Where indicated, cells were treated with AIIB2 (20 µg/ml), FGFRi (2 µM) and/or CDDP (0.5 µM).

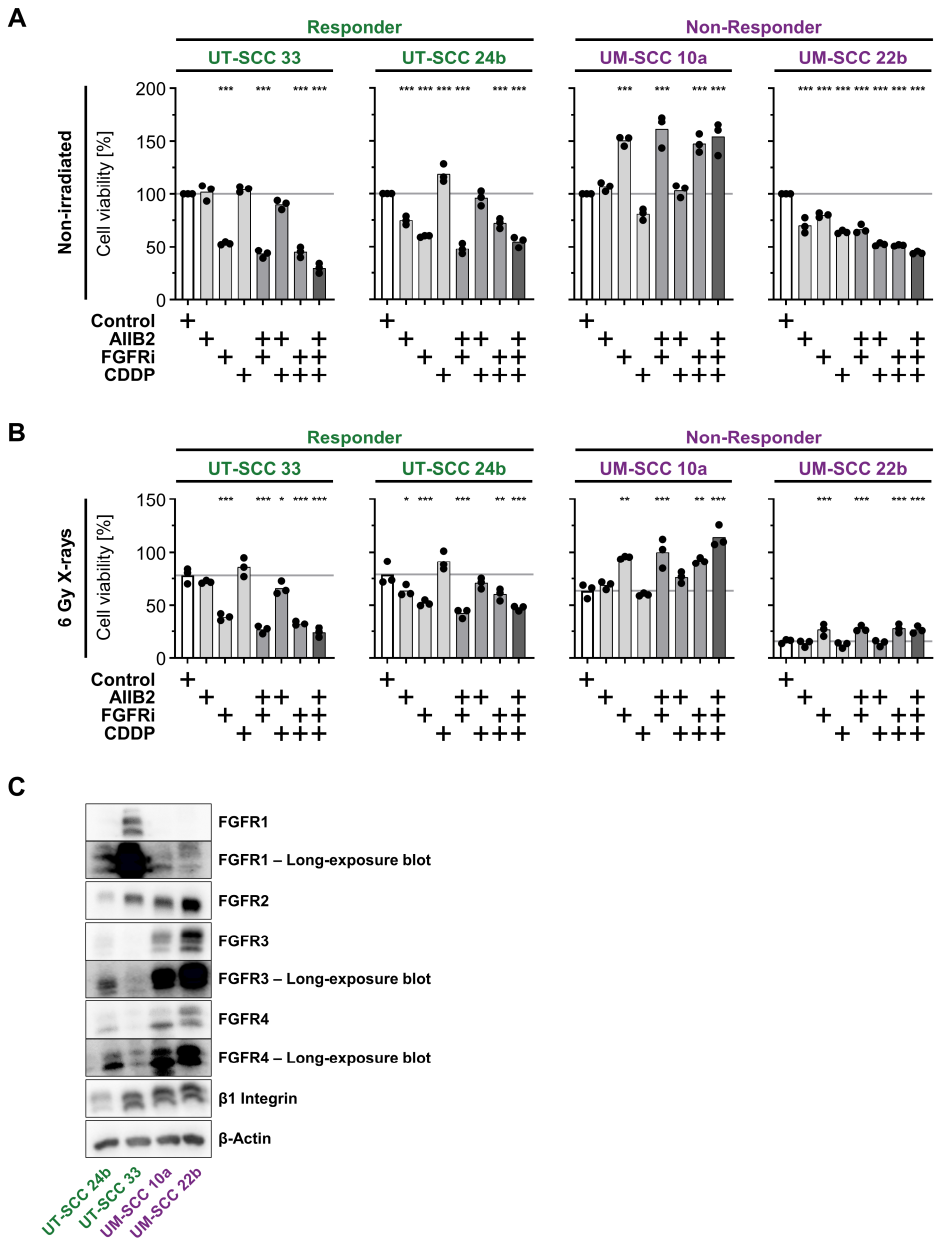

Fig. S4 Characterization of most sensitive and resistant cell models towards AIIB2/FGFRi/CDDP/irradiation reveals adverse effects. **A** Cell viability of indicated 3D lrECM grown HNSCC models upon indicted treatment combinations. DMSO/IgG were used as control (*n* = 3; mean; two-way ANOVA; Dunnett's multiple comparison test to corresponding controls; ***p ≤ 0.001; **p ≤ 0.01; *p ≤ 0.05). **B** Cell viability of shown 3D lrECM grown HNSCC models upon indicated treatment combinations plus a single dose of 6 Gy X-rays. DMSO/IgG were used as control (*n* = 3; mean; two-way ANOVA; Dunnett's multiple comparison test to corresponding

irradiated controls; ***$p \leq 0.001$; **$p \leq 0.01$; *$p \leq 0.05$). Where indicated, cells were treated with AIIB2 (20 µg/ml), FGFRi (2 µM) and/or CDDP (0.5 µM). **C** Western blot analysis of basal FGFR and β1 integrin expression from whole cell lysates of indicated 3D lrECM cell cultures. β-actin served as loading control. Representative blots from three independent experiments are shown, supplemented with corresponding long-exposure blots for low intensity markers.

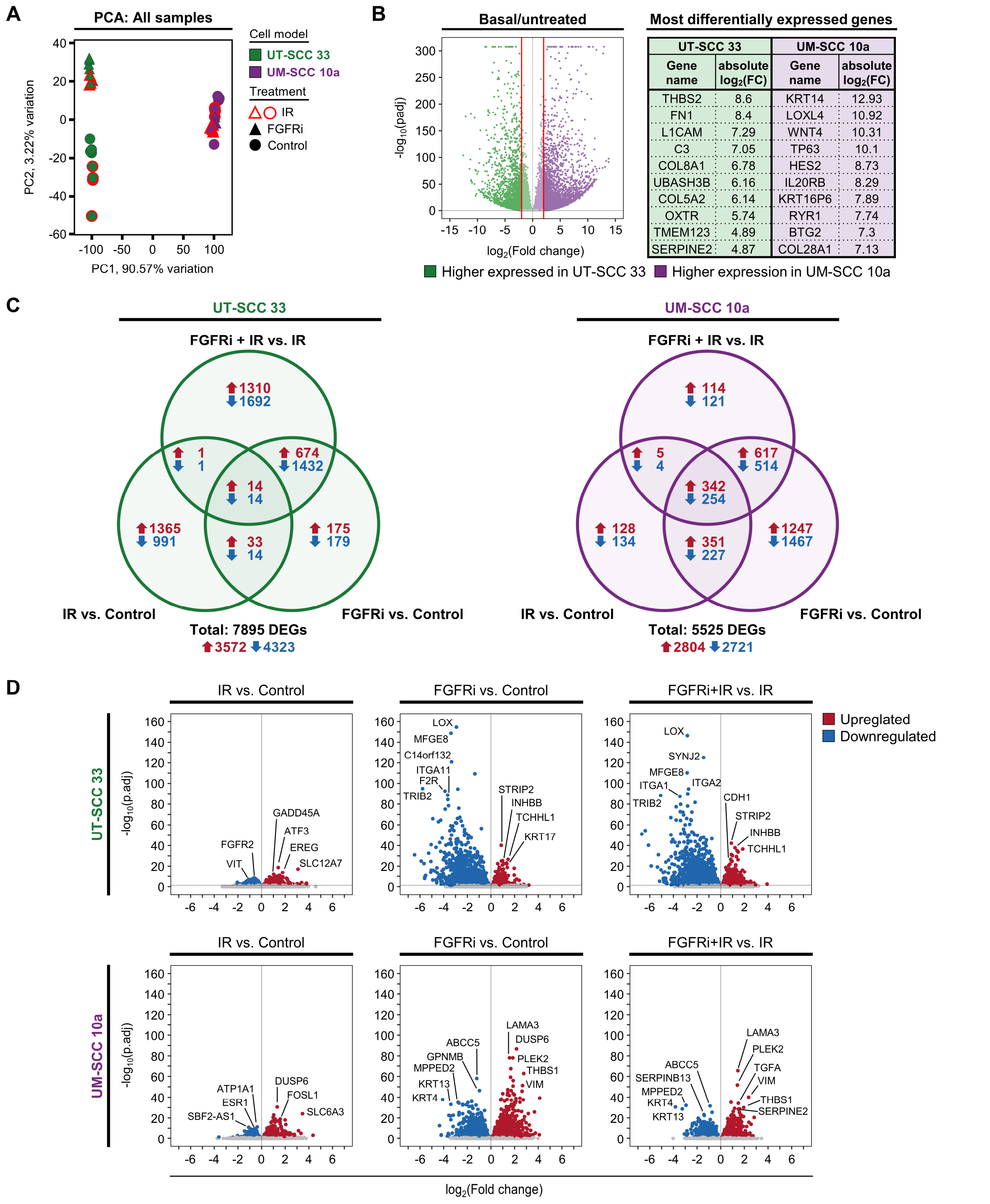

Fig. S5 Differential expressed genes of top FGFRi sensitive and resistant cell models delineate their intrinsic and induced response. **A** Principal component analysis of transcriptomic datasets

from all treated and untreated biological replicates across UT-SCC 33 and UM-SCC 10a cell models (*n* = 4). **B** Volcano plot of differentially expressed genes (DEG) between the UT-SCC 33 and UM-SCC 10a cell models at basal/untreated conditions (adjusted p-val. ≤ 0.05). The 10 most significantly altered genes are listed for each cell model in the adjacent table. **C** Venn diagrams showing the number of upregulated (red) and downregulated (blue) DEG (coding and non-coding) for each treatment-to-control comparison group in UT-SCC 33 and UM-SCC 10a cell models (IR, 6 Gy X-ray irradiated; FGFRi, FGFR inhibitor treatment; FGFRi/IR, combined treatment). **D** Volcano plots of the three DEG comparison groups (IR; FGFRi; FGFRi+IR) for UT-SCC 33 and UM-SCC 10a cell models. All DEG with adjusted p-value (p.adj) ≤ 0.05 are colored accordingly. Candidates from the top deregulated genes on both ends based on significance are indicated.

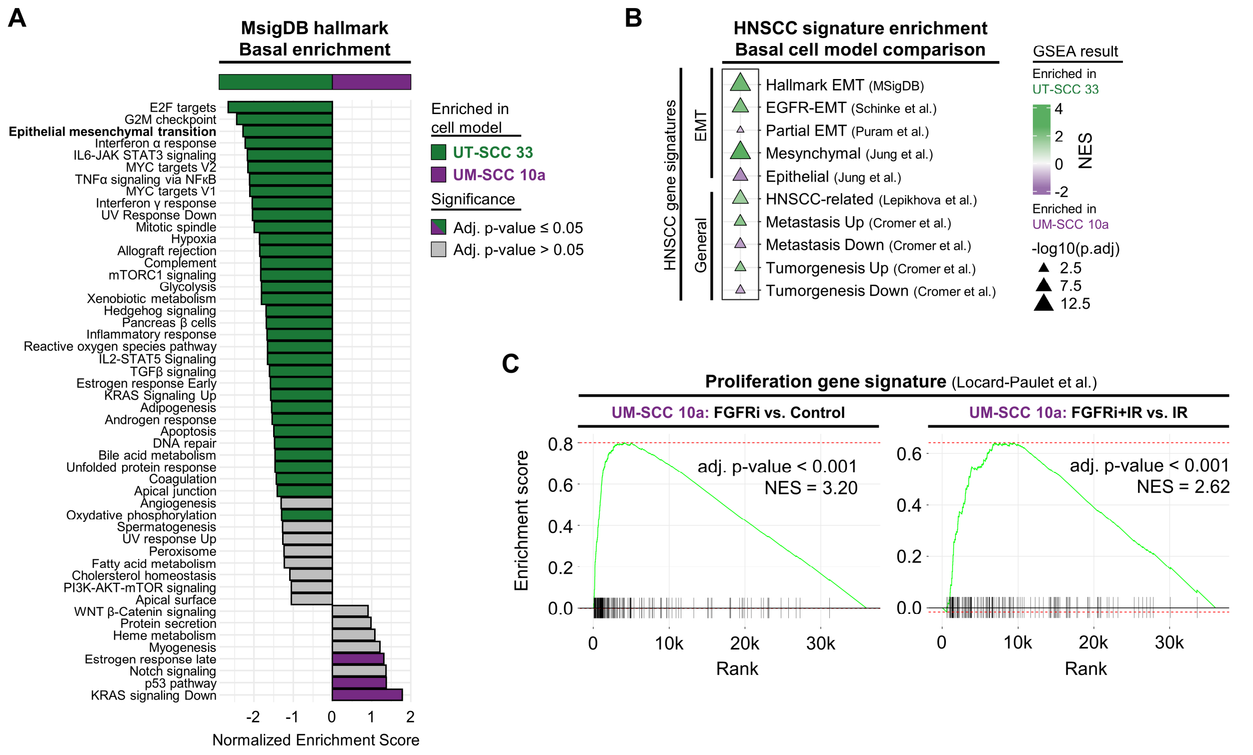

Fig. S6 Basal and treatment-induced changes of EMT markers pinpoint the importance of this cancer hallmark. **A** Deviating bar plot of MsigDB-hallmark gene set enrichment analysis (GSEA) between UT-SCC 33 and UM-SCC 10a cell models at basal/untreated conditions. Normalized enrichment scores (NES) and their corresponding significance (adjusted p-value ≤ 0.05) are depicted. **B** Normalized NES summary graph of multiple GSEA of indicated gene sets (**Table S3**) for basal/untreated UT-SCC 33 vs. UM-SCC 10a cell model comparison. Results are presented by NES in the color of the cell model in which the respective signature is enriched. Significance levels (adjusted p-value ≤ 0.05) are indicated by triangle size. **C** GSEA enrichment plots for a proliferation gene signature (**Table S3**) for the indicated treatment comparisons of the cell model UM-SCC 10a.

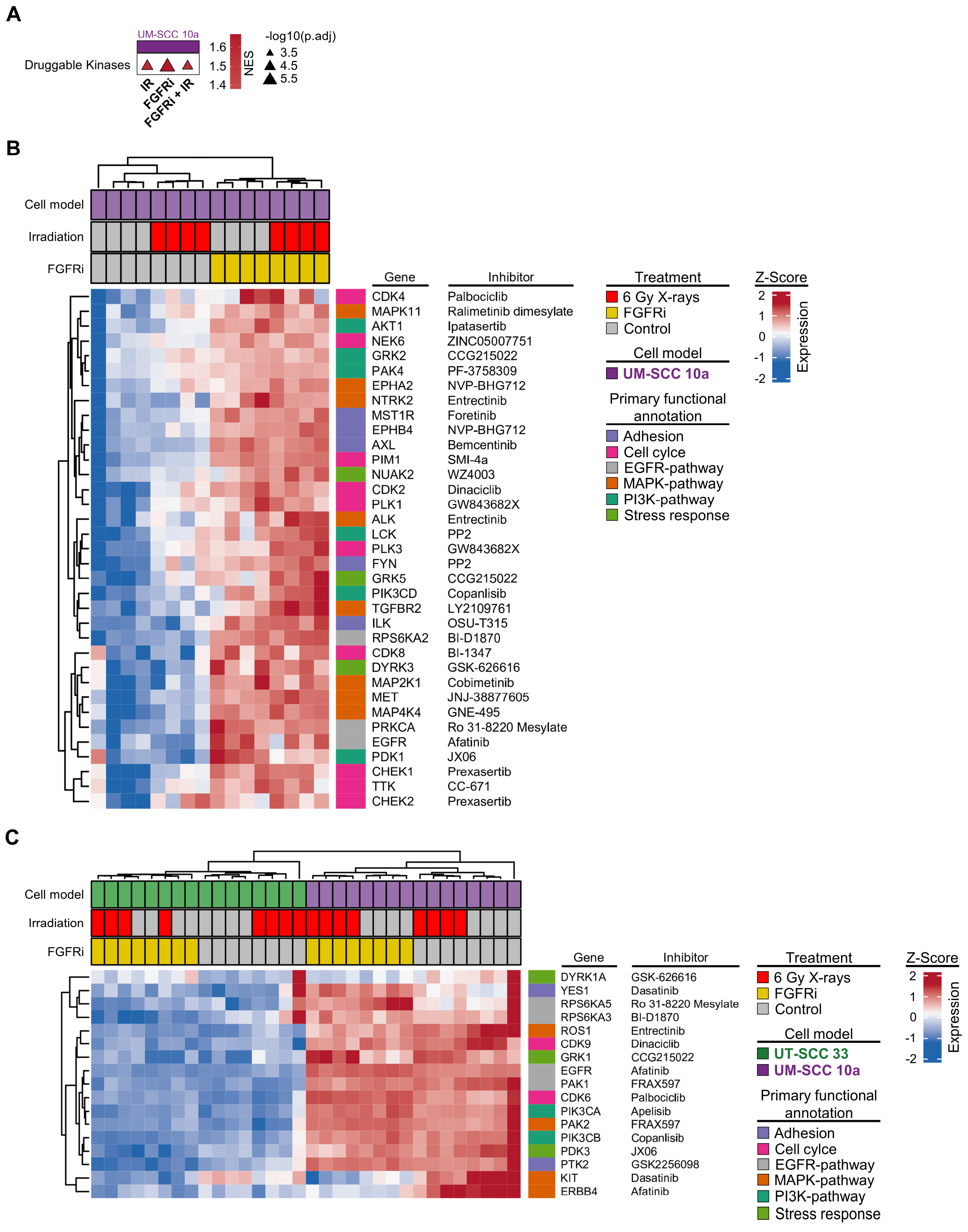

Fig. S7 Upregulated or strongly expressed kinases present promising candidates for specific pharmacological inhibitors to overcome FGFRi-induced resistance. **A** Normalized enrichment

score (NES) summary graph of three gene set enrichment analyses (GSEA) for druggable kinases (**Table S3**) in each DEG comparison group (IR, 6 Gy X-ray irradiated; FGFRi, FGFR inhibitor treatment; FGFRi/IR, combined treatment) in UM-SCC 10a cells. Significance levels (adjusted p-value ≤ 0.05) are indicated by triangle size. **B** Gene expression heatmap of significantly (adjusted p-value ≤ 0.05) increased druggable kinases upon FGFRi treatment in UM-SCC 10a cells. Columns represent biological replicates (n = 4), rows represent z-score normalized gene expression data, both clustered hierarchically. Applied corresponding pharmacological inhibitors are listed. **C** Gene expression heatmap of significantly (adjusted p-value ≤ 0.05) higher expressed druggable kinases at basal/untreated conditions in UM-SCC 10a relative to UT-SCC 33 cells. Columns represent biological replicates (n = 4), rows represent z-score normalized gene expression data, both hierarchically clustered including gene annotations to specific cell functions. Applied corresponding pharmacological inhibitors are listed.

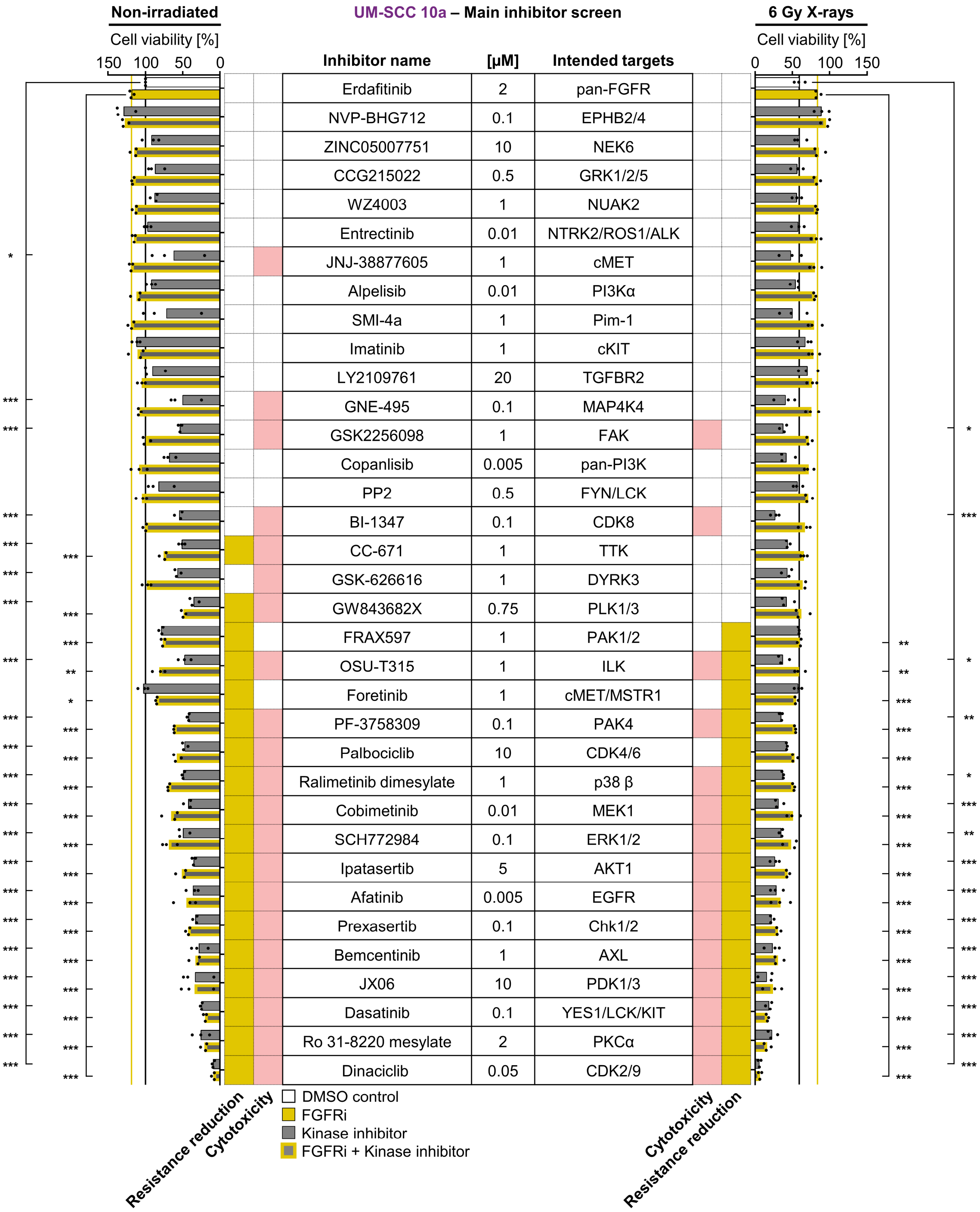

Fig. S8 Drug screen cell viability data with selected kinase inhibitors. Graph illustrating cell viability of non-irradiated (left panel) or 6 Gy X-ray irradiated (right panel) 3D lrECM UM-SCC 10a cell cultures treated as indicated. Bars display mean cell viability (*n* = 3; two-way ANOVA; Tukey multiple comparison test to corresponding non-irradiated (left panel) or irradiated (right panel)

controls; ***$p \leq 0.001$, **$p \leq 0.01$, *$p \leq 0.05$). Color annotation highlights inhibitor candidates which accomplished either a significant reduction of the FGFRi-induced resistance (yellow, i.e. resistance reduction) or a significant reduction of DMSO controls (red, i.e. cytotoxicity).

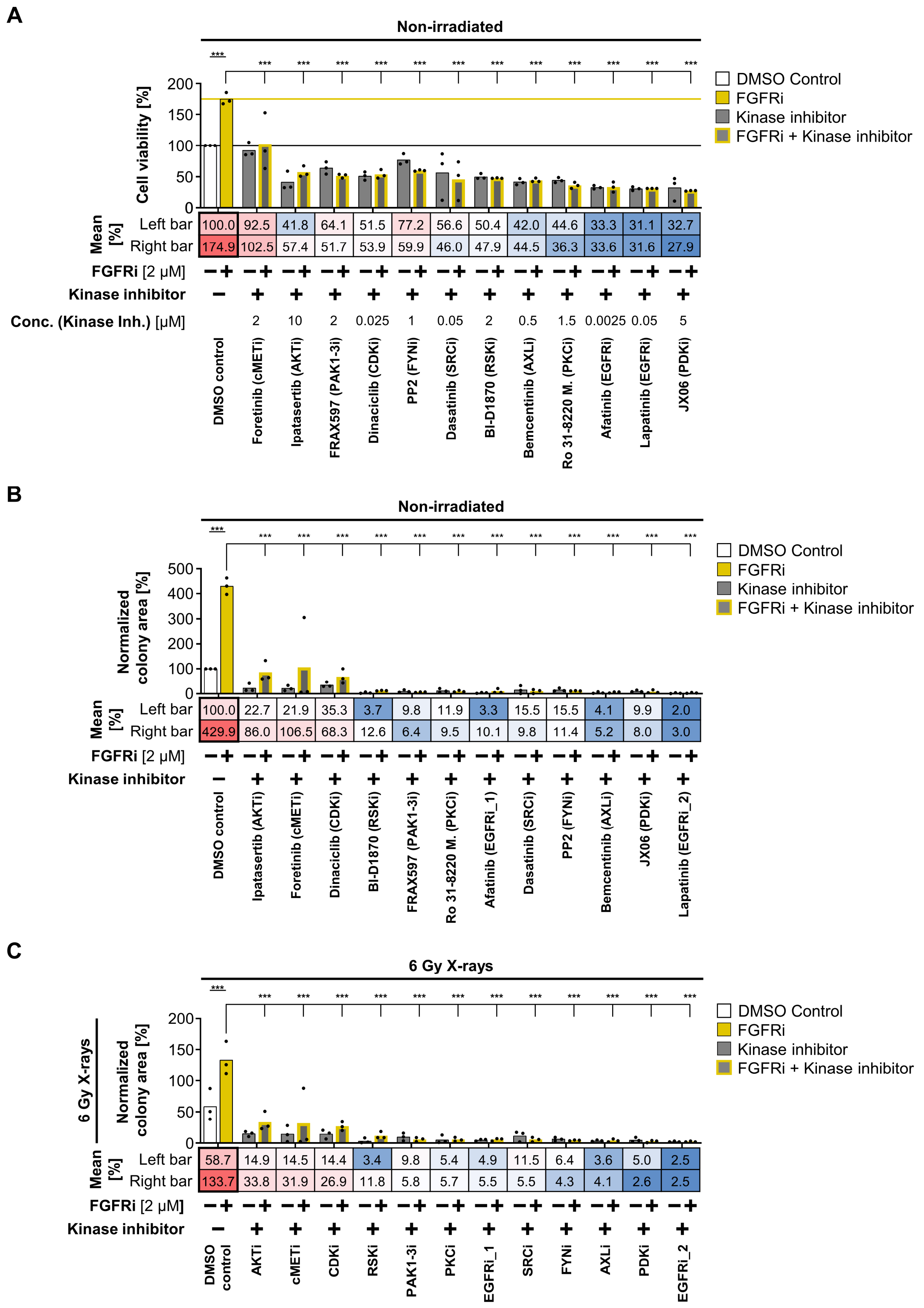

Fig. S9 Selected kinases deactivate FGFRi-induced resistance in UM-SCC 10a cells. **A** Effects of indicated kinase inhibitors on cell viability in UM-SCC 10a cells. Bars and the bottom annotation

table display mean cell viability (*n* = 3; two-way ANOVA; Tukey multiple comparison test; ***p ≤ 0.001). **B** Normalized colony area of 3D lrECM UM-SCC 10a cell cultures treated as indicated (inhibitor concentrations are listed in **Fig. S9A**). Absolute areas were normalized to the means of corresponding controls. Bars and the bottom annotation table display mean normalized colony areas (*n* = 3; two-way ANOVA; Tukey multiple comparison test; ***p ≤ 0.001). **C** Normalized colony area of 6 Gy X-ray irradiated UM-SCC 10a cells treated indicated inhibitors (concentrations as listed in **Fig. S9A**). Absolute areas were normalized to the means of the corresponding non-irradiated controls (**Fig. S9B**). Bars and bottom annotation table display mean normalized colony areas (*n* = 3; two-way ANOVA; Tukey multiple comparison test; ***p ≤ 0.001)**.**

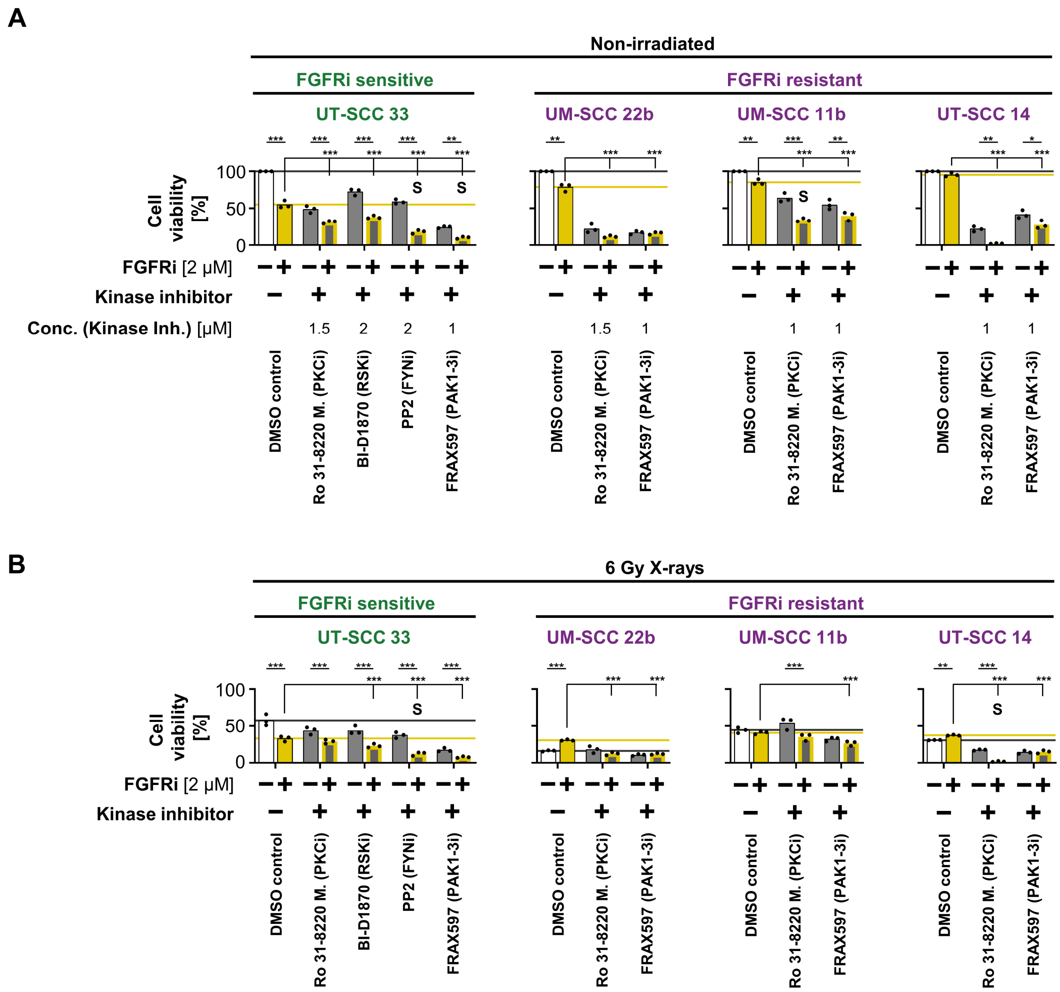

Fig. S10 Selected kinase inhibitors enhance FGFR targeting efficacy in sensitive and resistant HNSCC cell models. **A** Normalized cell viability of indicated non-irradiated cell models upon treatment with selected kinase inhibitors alone (left bar) or in combination with FGFRi (right bar). Applied kinase inhibitor concentrations are indicated. Bars represent mean cell viability (*n* = 3; two-way ANOVA; Tukey multiple comparison test; ***p ≤ 0.001, **p ≤ 0.01, *p ≤ 0.05). 'S' indicates synergy calculated by the Bliss independence model. **B** Normalized cell viability of indicated 6 Gy X-ray irradiated cell models upon kinase inhibitor monotherapy (left bar) or in combination with FGFRi (right bar). Applied kinase inhibitor concentrations are indicated in **Fig. S10A**. Bars represent mean cell viability (*n* = 3; two-way ANOVA; Tukey multiple comparison test; ***p ≤ 0.001, **p ≤ 0.01, *p ≤ 0.05). 'S' indicates synergy calculated by the Bliss independence model.

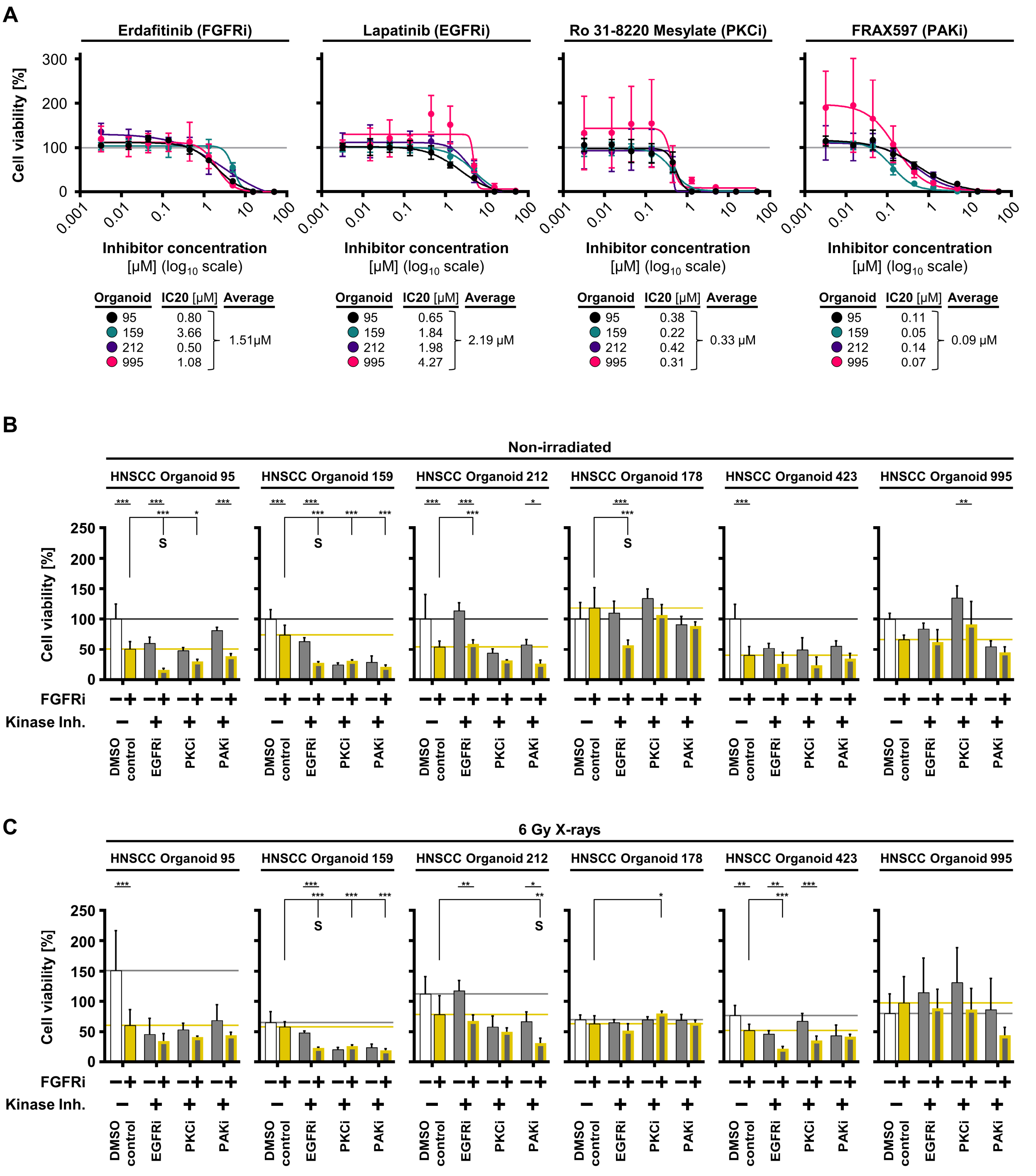

Fig. S11 HNSCC organoids validate combinatory effectiveness of selected kinase inhibitors with FGFRi. **A** Mean cell viability (± standard deviation) of HNSCC organoids to a concentration range of indicated kinase inhibitors. Per data point, three technical replicates were averaged and normalized to the corresponding DMSO controls. Non-linear regression was used for IC20

calculations and the approximate mean IC20 was implemented in following experiments. **B** Normalized cell viability of indicated non-irradiated HNSCC organoids upon treatment with selected kinase inhibitors alone or in combination with FGFRi. Bars represent mean cell viability (*n* = 6 technical replicates; two-way ANOVA; Tukey multiple comparison test; ***p ≤ 0.001, **p ≤ 0.01, *p ≤ 0.05). **C** Cell viability of indicated 6 Gy X-ray irradiated HNSCC organoids upon kinase inhibitor monotherapy or combination therapy with FGFRi. Bars represent mean cell viability normalized to the corresponding non-irradiated control (*n* = 6 technical replicates; two-way ANOVA; Tukey multiple comparison test; ***p ≤ 0.001, **p ≤ 0.01, *p ≤ 0.05). Where indicated, organoids were treated with 1.5 µM FGFRi, 2 µM EGFRi, 0.33 µM PKCi or 0.1 µM PAK1-3i (DMSO served as control). ‘S’ indicates synergy calculated by the Bliss independence model.

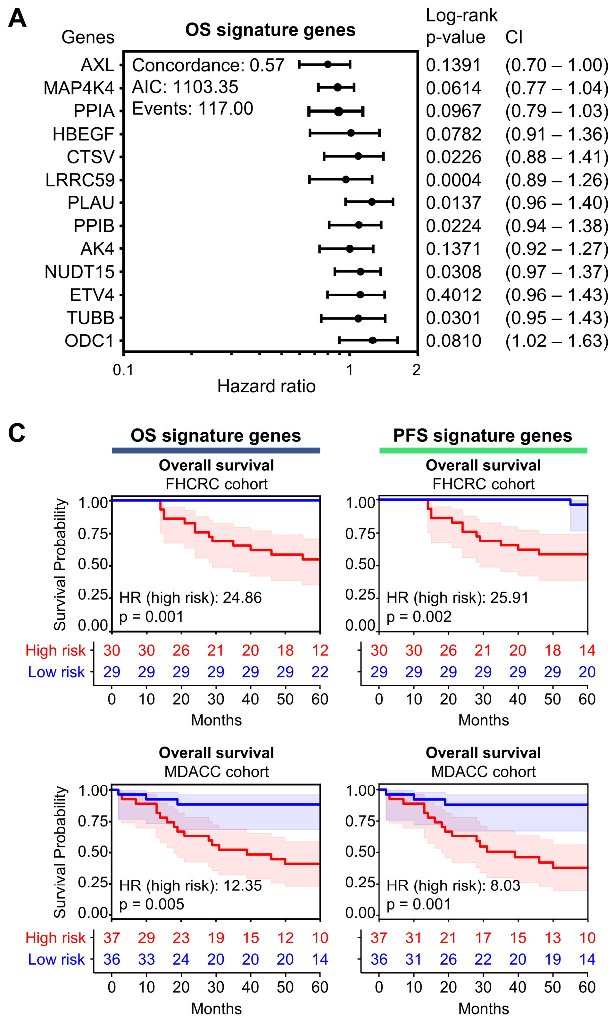

Fig. S12 Transfer of the transcriptomic in vitro FGFRi-resistance response signature to clinical cohorts yields relevant risk scores. **A** Forest plot of the multivariable Cox regression model incorporating overall survival (OS) risk score signatures into the HPV-negative HNSCC training cohort (TCGA, *n* = 415). Hazard ratios are plotted; concordance index, Akaike information criterion (AIC), event numbers, log-rank p-values and confidence interval (CI) are indicated. Additional information is provided in **Table S3**. **B** Forest plot of the multivariable Cox regression model incorporating progression-free survival (PFS) risk score signatures into HPV-negative HNSCC training cohort (TCGA, *n* = 415). Hazard ratios are plotted; concordance index, Akaike information criterion (AIC), event numbers, log-rank p-values and confidence interval (CI) are indicated. Additional information is provided in **Table S3**. **C** Validation of OS and PFS risk scores

in HNSCC cohorts of the Fred Hutchinson Cancer Research Center (FHCRC; *n* = 59) and the MD Anderson Cancer Center (MDACC; *n* = 54) for overall survival. Median cut-offs were used. Hazard ratio of high-risk patients (red curve) and log-rank test p-values for the comparison of high- and low-risk groups are indicated together with 95% confidence intervals in Kaplan-Meier curves including patient at risk numbers.

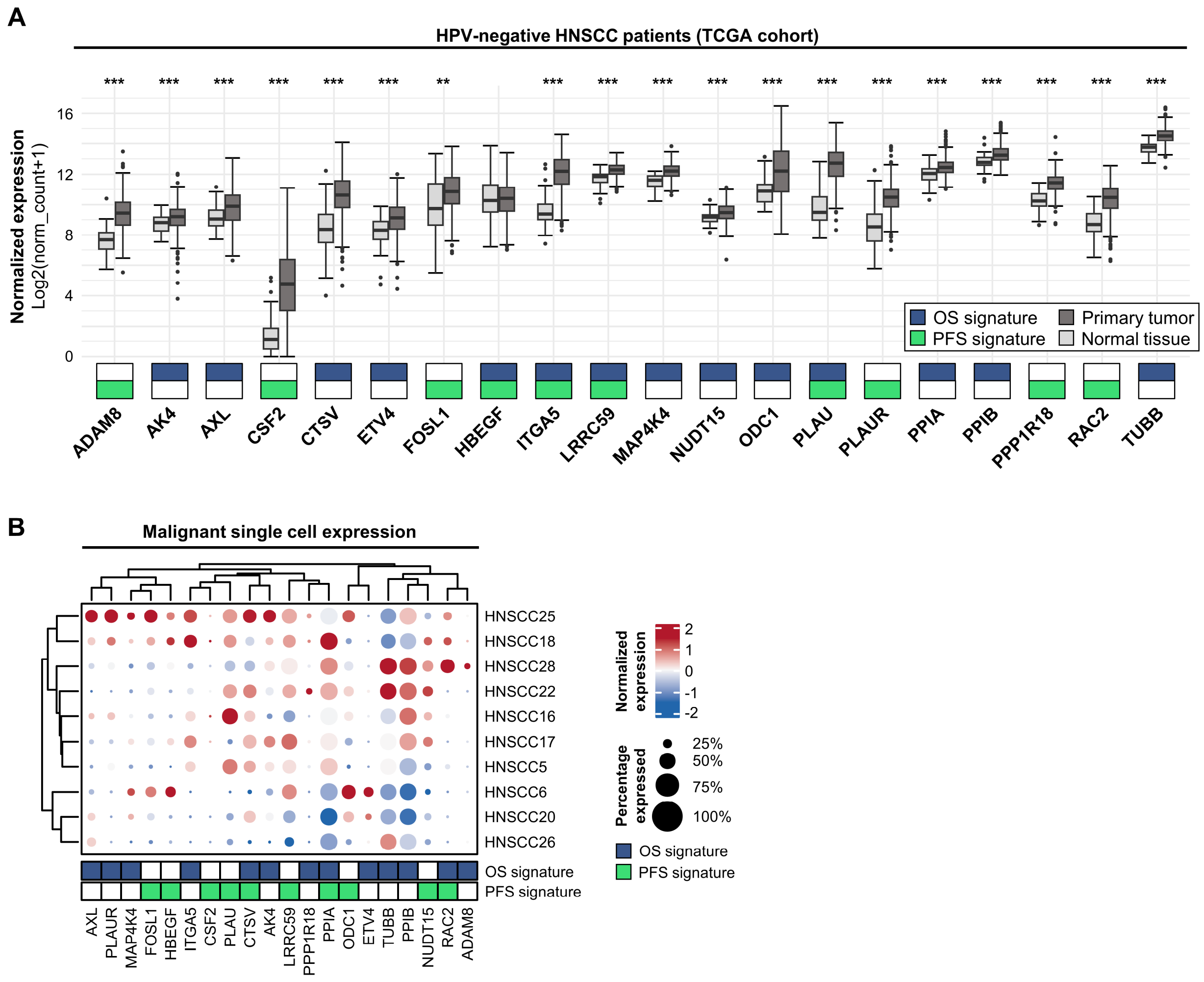

Fig. S13 Identified signature genes are expressed in both the HNSCC TCGA cohort and single HNSCC cells. **A** Gene expression analysis of the 20 OS and PFS signature genes in HPV-negative HNSCC patients of the TCGA cohort. Data of primary tumor (*n* = 415) and normal tissue (*n* = 44) were compared using unpaired t test (***p ≤ 0.001, **p ≤ 0.01, *p ≤ 0.05). **B** OS and PFS gene signature expression in single HNSCC cells (*n* = 1891) from 10 HNSCC patients (GSE103322). Circle size represents the percentage of cells with recorded expression of the respective gene per tumor; circle color code indicates the average expression values.

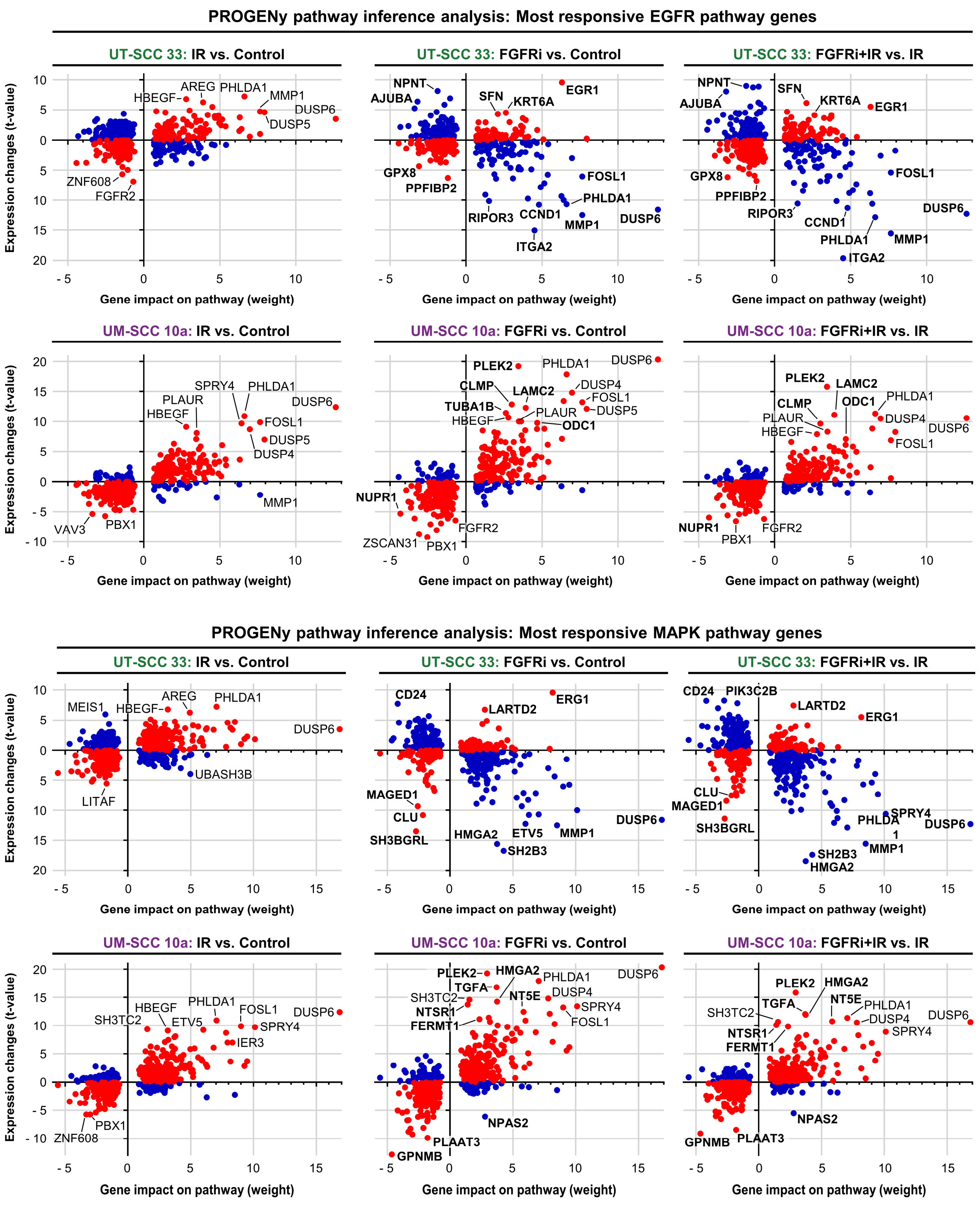

Fig. S14 PROGENy pathway inference delineates the most responsive EGFR/MAPK pathway genes upon FGFRi and irradiation. Gene-level PROGENy data for EGFR (upper panel) and

MAPK (lower panel) pathways are listed for the two cell models in the indicated treatment comparisons (IR, 6 Gy X-ray; FGFRi, FGFR inhibitor; FGFRi/IR, FGFR inhibitor/6 Gy X-ray). Each dot represents a gene with an assigned impact on the respective pathway (x-axis; weight), and the corresponding change in expression upon the indicated treatment (y-axis; t-value). Red dots represent an upregulation of the pathway (upregulation of positive-weight genes, downregulation of negative-weight genes). Blue dots represent a downregulation of the pathway (downregulation of positive-weight genes, upregulation of negative-weight genes). Bold gene names highlight candidates specifically altered in response to FGFRi.

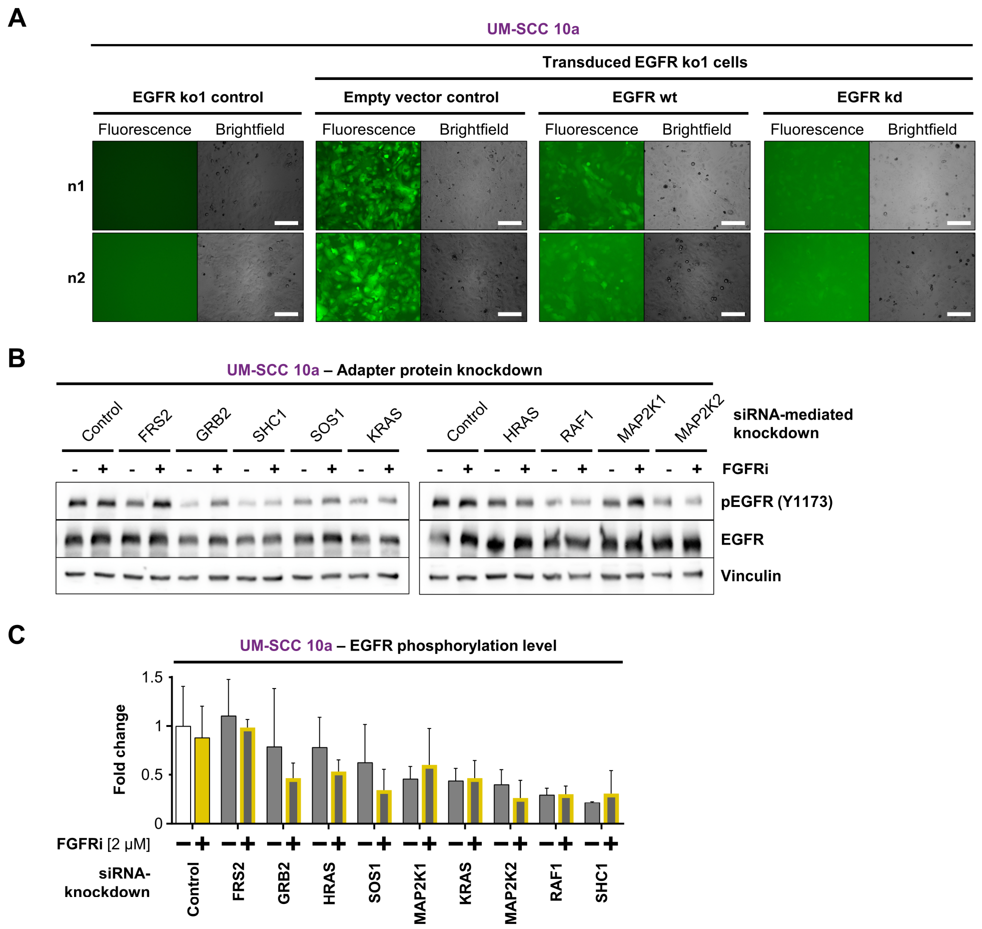

Fig. S15 EGFR phosphorylation is markedly diminished by depletion of specific adapter proteins of the EGFR or FGFR signaling cascade. **A** Representative images of UM-SCC 10a EGFR knockout cells (ko1) reconstituted with either EGFR wild-type (wt) or kinase-dead (kd) constructs. The desired cDNA is co-expressed with EGFP. Hence, positive fluorescence signals demonstrate construct expression. Empty vector construct served as transduction control, untreated EGFR ko1 cells were used to check for autofluorescence. Bar, 200 µm. **B** Western blot analysis of phosphorylation level (Y1173) and total EGFR expression in whole cell lysates from UM-SCC 10a cells upon siRNA-mediated knockdown of indicated target genes alone or in combination with FGFRi treatment (DMSO and non-targeting siRNAs served as controls). Vinculin served as loading control. Representative blots are shown. Where indicated, cells were treated with 2 µM FGFRi. **C** Densitometric analysis of western blot data shown in 'B'. Mean fold changes (± standard

deviation) compared to the corresponding controls are shown (*n* = 3). Only non-FGFRi treated densitometry data (grey bars) were used in further analysis (**Fig. 8l**).

Additional file 2: Table S1

Supplementary materials: Cell models, HNSCC organoids, Inhibitors, Western blot antibodies, Primers.

Additional file 3: Table S2

Supplementary information: FDA-approved inhibitors, Selected whole exome sequencing mutations.

Additional file 4: Table S3

Supplementary RNA-seq information: Overrepresentation analyses (ORA), Gene signatures, Differential expressed genes (DEG) data with relevant annotations. Supplementary results for Cox regression models.
